# Citizen science and social innovation as citizen empowerment tools to address urban health challenges: The case of the urban health citizen laboratory in Barcelona, Spain

**DOI:** 10.1371/journal.pone.0298749

**Published:** 2024-03-13

**Authors:** Celia Santos-Tapia, Matias Verderau, Sílvia Borràs, Marta Flórez-Santasusana, Francisco Flórez, Juan José Morales, Pere Moli, Andrea Borràs, Marta Cirach, Mònica Ubalde-López

**Affiliations:** 1 Barcelona Institute for Global Health (ISGlobal), Barcelona, Spain; 2 Associació Lichen Innovación Social (Lichen IS), Barcelona, Spain; 3 Associació Trinitat Uneix, Barcelona, Spain; 4 Universitat Pompeu Fabra (UPF), Barcelona, Spain; 5 CIBER Epidemiología y Salud Pública (CIBERESP), Madrid, Spain; University of Coimbra: Universidade de Coimbra, PORTUGAL

## Abstract

Urban health faces significant challenges due to the rapid growth of cities and the concentration of population in urban settings that have a strong impact on people’s health. The approach to characterize and address these challenges requires increased societal involvement and interdisciplinary solutions to ensure their effectiveness and democratic nature. With this purpose, it is necessary to explore methodologies for citizen participation that foster a critical understanding of the environment and promote their active role in generating scientific knowledge and change. This article describes the creation of a collaborative space for experimentation and learning that, through the intersection of citizen science and social innovation, aims to engage citizens in the research and diagnosis of their local environment, as well as in the design and implementation of local solutions, while raising awareness about the main challenges to urban health. Through a collaborative and participatory framework, the community identified relevant challenges to urban health they wanted to investigate, co-designed and developed the methodology for data collection and analysis, and ultimately, they devised, designed, and implemented innovative solutions based on the scientific evidence obtained. The framework and results of this project hold potential interest for the scientific community, facilities, institutions, and society by offering an innovative and participatory approach to addressing the present and future urban health challenges.

## Introduction

Nowadays, over 55% of the world’s population live in urban areas and this proportion is expected to increase to 68% by 2050 [1]. This surge in global urbanization offers potential benefits, yet it can also introduce detrimental impacts on community health, due to the considerable influence that the organization of cities exerts on overall well-being. In this context, urban health allows us to understand how different characteristics of the urban environment (physical, social, and structural) can influence people’s health and disease [2]. As an example, exposure to high levels of particulate matter, noise, high temperatures and lack of access to natural areas cause a significant percentage of annual deaths, burden of disease and increased public expenditure on health due to various impacts on human health [3–5]. If we focus on the city of Barcelona (Spain), it is estimated that around 700 premature deaths occur each year due to these previously mentioned factors [6]. It is also known that these deaths could be prevented with a better urban planning [7, 8] and, for that reason, it is important to guide urbanization in a way that protects and promotes human health [9]. Considering this influence on the well-being of individuals, it is important that citizens possess the entitlement to receive information, voice their perspectives, and participate in decisions pertaining to factors that affect urban planning [10, 11]. In fact, there are already numerous initiatives led by different communities that are calling for better air quality and a healthier and more sustainable public space in cities [12–15]. Providing platforms for discourse and research, enabling affected citizens to actively participate and contribute is highly desirable for dealing with social complexity and address these global challenges [16, 17].

The contribution of citizens to improving urban health can take many forms, with one of them being citizen science (CS), an approach that has been gaining popularity in recent years [18]. CS can be defined in multiple ways, but a more inclusive and general description would be the active involvement of the public in scientific research to support knowledge generation [19–21]. Over the last few years, several CS projects have addressed issues such as well-being and livability [22], air quality [23–25], urban mobility [26], disease control [27], urban nature [28, 29] or healthcare [30]. In CS projects, citizen involvement ranges from occasional to more comprehensive, presenting a gradient of modalities in accordance with the degree or type of contribution and the goal they seek to achieve [31]. Some of these projects are designed by professional research teams and require citizen participation in specific tasks such as data collection or analysis, sharing their acquired knowledge, or providing their tools and resources for research purposes [21, 32–35], while others are designed collaboratively through mutual involvement of various stakeholders (co-created), and participants can be involved throughout all research phases [33, 36]. If the project prioritizes the interests of the participating community, the co-created approach promotes a shift from the traditional hierarchical scientific paradigm [37, 38] and allows for better outcomes, such as a more significant social impact, better scientific understandings, success in affecting on timely policy decisions or enhancement on resource management capacity of communities [39, 40].

Nowadays, the increasing use of technology to involve citizens in science provides new opportunities, such us gathering data beyond the scientific realm (e.g., gender and level of education of the participants) that can be valuable at the social and political levels (e.g., which audience might be more inclined to participate in CS projects and which audience would require specific actions to increase their willingness to engage) [41]. CS also offers a collaborative space between disciplines, being data science a good example as it can contribute to a wide range of disciplines in humanities, natural and social sciences, helping to enhance the quality of generated information (e.g., it aids in eliminating known biases or detecting malicious behaviours or data sabotage) [42]. Mentioning some challenges, the growth of participatory science today seeks strategies to motivate and/or reward participants, or debates on the ethical treatment of contributors [43].

CS has the power of providing new scientific evidence and an in-depth understanding of an issue, fostering increased scientific literacy or behaviour change. However, several case studies have noted that a common disappointment in CS projects is the lack of impact when participants contribute solely by generating evidence and data [44]. In that sense, it is important to complement research projects with spaces that allow for debate, formulation, and design of possible solutions to the investigated problem. In this regard, Living Labs [45] or Citizen Labs [48] offer a potential possibility to address this matter. These are open innovation ecosystems where individuals, communities, and organizations with different backgrounds in culture, belief, and knowledge, collaborate and experiment to achieve a common goal, leveraging local knowledge and experiences to develop tailored responses to specific needs [46]. These platforms provide space for what is called Social Innovation (SI), a practice through which participants develop and implement long-lasting solutions to meet a social need, improve quality of life, and promote positive change [47, 48]. In relation to the subject at hand, there are previous experiences where Living/Citizen labs, through social innovation, address different urban issues [49].

Combined, CS and SI can significantly enhance society’s ability to address complex challenges, synergizing effectively [50]. Indeed, CS, being a novel approach, might be considered as SI in the realm of the traditional research process, and CS might be treated as a vehicle to foster SI [34]. The power of combining both approaches lie in the ability to leverage scientific rigor to explore issues within our environment, while providing with the opportunity to translate that research into action, making a positive impact on the community [47] and generating new social structures and relationships that increase its capacity to act [53]. However, to the best of our knowledge, there are currently little synergy experiences between CS and SI to address urban health problems [51–53], and none of these experiences include a co-created CS approach.

Citizen’s Urban Health Laboratory (CSU LAB by its Catalan name) is an urban health project funded by *Fundació BIT HABITAT* (Barcelona’s City Council) under the *Ciutat Proactiva* call. It proposes a pilot participatory methodology to integrate co-created CS with the SI approach of Citizen Labs. The main aim of the project is to create a collaborative space of research and practice to encourage community participation in shaping the research on urban health and promoting more resilient, healthy, sustainable, and inclusive neighbourhoods. The laboratory is articulated in 4 phases: (i) a participatory diagnosis to define problems of interest to citizens, (ii) a CS process for participatory research, (iii) a SI phase to propose solutions, and (iv) a communication phase to disseminate the results. The modular methodology used in CSU LAB allows participants to join any of the phases, either generally or specifically, considering their diverse availability and interests, ensuring a flexible and inclusive space for participation. In this paper, we describe the participative process and results obtained from the first experience of the project conducted in the neighbourhood of Trinitat Vella, in the city of Barcelona (Spain). As a result, participatory research on the air quality and noise in the neighbourhood was co-developed and two innovative solutions proposed by participants—an educational environmental suitcase and an urban intervention signage kit—were prototyped and implemented in the local context.

## Materials and methods

**Fig 1:**
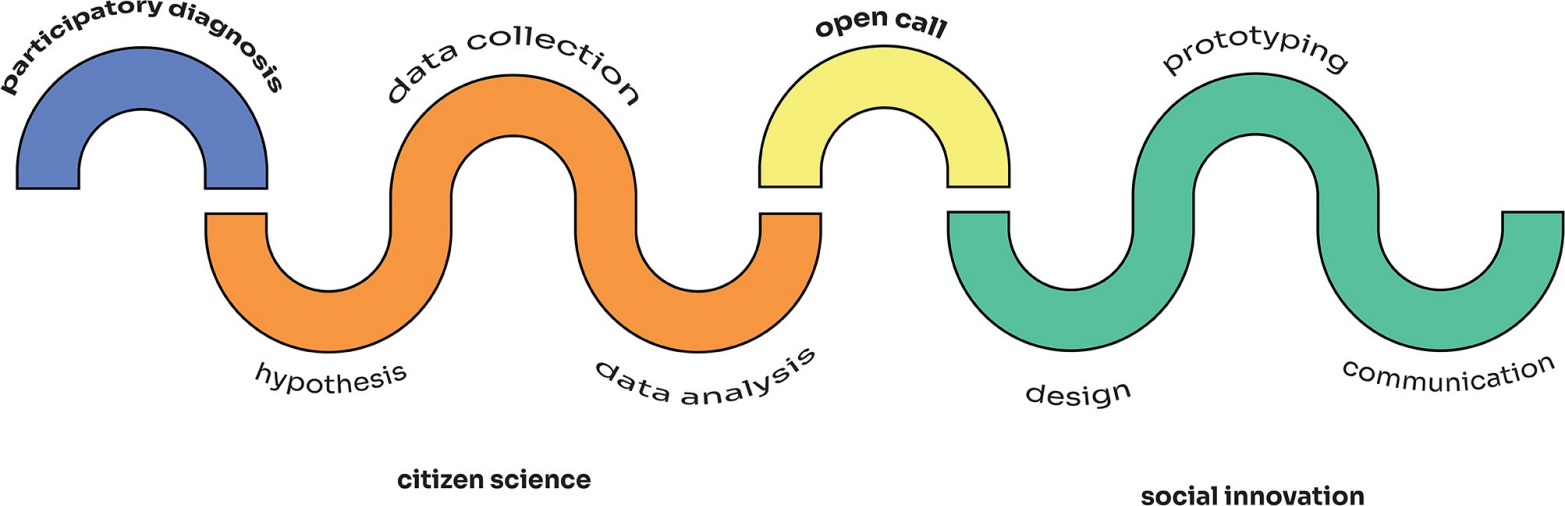
LAB CSU methodological framework. The methodological framework consists of a first phase of participatory diagnosis, a second phase of citizen science (which includes the design of the hypothesis and research question, data collection, and data analysis), and a third phase of social innovation (which encompasses the design and prototyping of ideas and communication).

The methodological framework of the CSU LAB aims to combine CS with SI. On the one hand, the innovation phase is based on the previously described approach of *Distributed Citizen Laboratories*, a methodology promoted by MediaLab Prado that seeks to create diverse and inclusive spaces that facilitate collaboration among people to experiment, design, and implement projects that improve urban life [38] promoting the active role of citizens as producers of ideas in their local environment [54]. This innovation methodology is primarily focused on collaborative prototyping, with a special emphasis on the preparation, production, and post-production processes. On the other hand, a basic framework to develop a CS project was designed, encompassing all the diverse stages of a research process and drawing [23, 55]. As a result, the project is based on a 4- stage process, as follows. First, it started with a participatory diagnosis to gain insights into the topics of interest that citizens want to investigate. Using this information, a CS process was developed, in which the research question and hypothesis were co-defined, the research work was co-organized (i.e., determine the variables to measure to answer the research question, select the tools to use, decide when and where to collect the data and who is going to do it), and the data collected was co-analyzed. Based on the obtained results, an open call for proposals collected ideas for social innovation that included different steps: selection, design and prototyping. A final communication phase aims to enhance the project’s impact and promote the appropriation of the results by the entire participating community. A visual outline of the framework can be found in Fig 1.

As shown in the methodological framework, each phase is divided into several steps, which in turn have different objectives. The project is mostly implemented on a neighborhood scale to work on feasible and local solutions with the community. However, certain parts of the project, such as the participatory diagnosis or the open call, are carried out at the city level to gather a greater diversity of ideas, proposals and sensitivities that can enrich the overall process. Details of the steps, objectives, tools used, and the scope of each phase are shown in Table 1. Their detailed use will be outlined in the different sections.

**Table 1:** The methodological framework of CSU LAB.

Step	Aim	Tool	Scope
**Set up: Neighbourhood selection and participant recruitment**			
Neighbourhood selection and participant recruitment	To discuss and evaluate the feasibility of the project implementation in different districts with administration representatives and to recruit participants	Meetings and informal conversations	City
**Phase I: Participatory diagnosis**			
Diagnosis	To identify topics citizens are concerned about and would like to investigate in the context of urban health	Participatory diagnosis canvas (Kick-off session)	City
		Online survey	City
		Participatory diagnosis canvas	Neighbourhood
**Phase II: Citizen Science**			
Definition of the research question and hypothesis	To deepen into the identified problematics	Problem definition canvas	Neighbourhood
	To select the research question	Hypothesis and research question definition canvas	
Definition of the data collection protocol and process	To define the study protocol and data collection tools	Work organization canvas	Neighbourhood
	To collect the data	Smart Citizen Kit and Palmes diffusion tubes	
Data analysis	To analyze and discuss the data	Open discussion	Neighbourhood
**Phase III: Social Innovation**			
Open call and proposals selection	To collect and select two citizen proposals to innovate in urban health	Online survey and pop-up intervention	City
Proposals design	To design the selected prototypes	Knowledge mapping, prototype ideation and prototype planning canvas	Neighbourhood
Proposals prototyping	To prototype the proposals	Digital fabrication tools, hand tools and collaborative tools.	Neighbourhood
**Phase IV: Communication**			
Communication	To design the communication activities and implement the prototypes	Communication plan canvas	City
**Evaluation**			
Evaluation	To evaluate the project	Evaluation survey	Participants

Detail of the aim, tools used and scope of the different phases and steps.

### Set up: Neighbourhood selection and participant recruitment

The first step in the project was to select a neighbourhood for its implementation. This part was carried out by the driving group, composed of researchers from a health research institute and from an organization specialized in social innovation. This driving group organized different meetings and informal conversations with representatives of the administration and organizations to discuss and evaluate the feasibility of the project implementation in different districts. The discussions started with four stakeholders in the administration within the fields of ecology, urbanism and mobility, citizen participation and community work at the city level (steps 1 and 2—S1 Table). We prioritized these administrative departments as essential to collaborate with to effectively structure a participatory project in the field of urban health. During these meetings, they helped us to identify neighborhoods o districts where the project could be relevant and connected us with four key stakeholders in these neighborhoods/districts (step 3—S1 Table). In a second round of meetings, these other local stakeholders helped us assess the viability of the project in different neighborhoods or districts, allowing us to focus on one, enabling collaboration with key social stakeholders and aligning the project with ongoing initiatives. Based on the conclusions of these meetings, the driving group finally focused on one specific neighbourhood (Trinitat Vella) (step 4—S1 Table) and contacted diverse grassroots organisations. The neighbourhood of Trinitat Vella was identified as the most suitable for implementing the project due to its urban reality (surrounded by major traffic routes and highly vulnerable to heat) and social reality (socio-economic vulnerability but with a long history of participation). In Trinitat Vella, contact was established with Trinitat Uneix, a neighborhood association that focuses on environment, urban planning, and heritage. The project information and invitation to participate were provided to this entity both in written form (via email) and orally (through a meeting). Members of Trinitat Uneix showed interest in participating and promoting the project in the territory and eventually formed the core participants of the project. From that moment, they participated in all project activities. Additionally, an open call was made at each new phase of the project to allow anyone interested to join in any phase, including project design and data collection. This contact was made through official websites, social media platforms and/or neighborhood signage. These measures enabled individuals from outside Trinitat Uneix to become part of the project.

The modular nature of the project framework allowed participants to join specific phases without the need to commit to the entire process. Citizen participation served a dual role: providing their opinions and experiential knowledge to define the research process itself (e.g., defining the research question) and assisting in data collection. No personal or sensitive data was collected, and the information provided by the participants during the sessions was collected in an irreversibly anonymous manner. Information about the purpose of each phase and the co-creation nature of the project was provided before the sessions (through communication materials such as infographics or social media posts) and during the sessions both orally and in writing through presentations.

### Phase I: Participatory diagnosis

The objective of the participatory diagnosis was to identify topics citizens are concerned about and would like to investigate, both at the city’s and the neighbourhood’s level, in the context of urban health.

At the city level, we invited different stakeholders in the field of education, mobility, urbanism, and participation to a kick-off meeting. These stakeholders were part of the contact network of the driving group, with whom previous work had been done or who were key in the fields mentioned before. A total of 130 stakeholders were directly invited by e-mail, and the invitation was also openly shared through social media. The aim of this event was: (1) to present the project to the stakeholders, (2) to give an informative talk on urbanism and health and (3) to make the participative diagnosis itself. For the participatory diagnosis, we designed and used a canvas that consisted of seven questions to collect information on what aspects of urban planning are considered to be most important for health, which populations are considered vulnerable and possible research topics. The canvas was placed on one wall of the room and participants marked their answers freely using stickers. The participatory diagnosis canvas can be found in S1 Fig. In addition to the kick-off meeting, we also developed and distributed an online survey that contained the same questions as the participatory canvas. The survey was distributed via social media and, in addition to providing data for the diagnosis in a broader context, it also helped us to raise awareness of the project among the public. The specific questions of the survey can be found in S2 Table. In both cases (the questions from the canvas and the survey), they were based on existing previous evidence about risk factors in urban health and the already described vulnerable populations [56, 57].

At the neighbourhood level, we organized the first meeting with the community, through a grassroots organisation that agreed to promote the project in the territory. The aim of the session was to introduce the project, raise awareness about the impact of urbanism on human health among participants and carry out the diagnosis at the local level. In addition to the canvas used in the kick-off, we designed and used two extra participatory canvas: one to reflect on the individual’s impact on the city, and another to reflect on the impact of urban planning on participants. The canvas used can be found in S2 Fig. Finally, a collective mapping of relevant elements in relation to urban planning was organized.

### Phase II: Citizen science

The aim of the CS process was to conduct research on urban planning and health in the selected neighbourhood in a cooperative and participatory manner. We divided the process into three different parts: (1) the definition of the research question and hypothesis, (2) the definition of the data collection protocol and process, and (3) the data analysis.

(1) For the definition of the research question and hypothesis, we analyzed the data obtained in the diagnosis phase and presented it to the participants. This data was a starting point for the discussion to define a research question that was guided by using a problem definition canvas, and a hypothesis and questions definition canvas. The problem definition canvas consisted of a poster with six sections: problem, evidence, causes, impact, affected population and others. We explained the canvas to the participants and guided a discussion with the group to deepen their understanding of the problems they identified in the diagnosis. After that, we used the hypothesis and questions definition canvas, which consisted of a poster with three sections: summary of the problem, research question and working hypothesis. This canvas was designed to drive the discussion and information gathered in the previous canvas to define a research question and a working hypothesis. All participants worked together in a plenary session. The template of the canvas can be found in S3 Fig.

(2) The aim of the definition of the data collection protocol and process session was to discuss the different variables that could be measured to answer the research question, to choose the tools for taking the measurements and to organise the work. First, a debate with participants was held to discuss different aspects of air quality, (e.g., particulate matter (PM), gaseous pollutants, etc.) and noise. Different measurement tools were also introduced (e.g., NO_2_ passive diffusion tubes, Arduino technology sensors for measuring temperature, noise and PM, mobile applications for measuring noise levels or sensors for vehicle counting). The mapping of these possible tools was carried out once the diagnosis was completed and before defining the research question. The promoting team conducted a preliminary search for tools that could be used to address the topics of interest, mainly based on those that had been successfully used in previous projects. These tools were either available at the research center or could be purchased/requested within a time frame compatible with the project. Only those tools related to the research question ultimately chosen were presented. The option of proposing new tools was also raised, but no proposals were made by the participants. For the definition of the data collection protocol and process, work organization canvas was used. This canvas consisted of a poster with seven questions: What is to be measured? Where will it be measured? Which tool will be used? Which day or days? At what time? For how long? Who will do the measurement? According to the selected research question, a discussion was driven with participants with the aim of selecting what variables they wanted to measure, choosing measurement tools, and scheduling the work. Participants selected those tools (from the previous selection) that were most practical or most interesting to use in the present project. The template of the canvas can be found in S4 Fig.

Among the proposed tools, participants chose to measure ambient NO_2_ by passive sampling with Palmes diffusion tubes, and noise and particulate matter pollution (PM) using Smart Citizen Kits (SCKs). Palmes sampler consists of an acrylic tube 7.1 cm long and 1.1 cm internal diameter, two stainless steel grids and two caps. A chemical reagent (triethanolamine) is used to absorb the pollutant directly from the air. In total, participants installed 14 Palmes tubes in the streets, approximately 1.5–2 m above the ground for 7 or 16 days. After the sampling period, the tubes were collected and sent to the laboratory for analysis. As a negative control, a blank tube (not exposed to ambient air) was also analysed. The SCK is a set of modular hardware components aiming to provide tools for environmental monitoring, ranging from CS and educational activities to more advanced scientific research. The SCK measures air temperature, relative humidity, noise level, ambient light, barometric pressure and PM. More information about the hardware of the SCK can be found on their website. Three participants took an SCK and installed it at their home windows/balconies (outside) for 9–18 days. A reference to the specific locations where NO_2_ and particulate matter measurements were taken can be found in S3 Table.

(3) Last, the data analysis session aimed to share the obtained results with participants and co-elaborate on the conclusions and further steps. For that, an open discussion with participants and some political stakeholders was organized. A brief report with tables, graphics and illustrations was presented during the meeting to show the data in a simple way and encourage discussion with the participants.

### Phase III: Social innovation

The social innovation phase aimed to design and implement methodologies and processes that favour the design and implementation of local proposals to work on the problems detected and investigated in the CS phase (phase II). Once the results of the diagnosis and CS in the territory were analyzed, the social innovation phase started. First, a call for ideas was open, where organizations and citizens made proposals for innovation in relation to the data obtained. The collection of proposals was done through two different tools: an online form that was disseminated through social networks, and an interactive station in the street, which was installed on the occasion of the Park(ing)Day initiative at the most polluted area of the neighbourhood, according to the CS data. The survey was open for 10 days and the Park(ing)Day activity lasted one day. After the established deadline, a jury of three people (one scientific researcher, one neighbour and one representative of the funding organisation) selected two proposals according to the evaluation criteria (that was published together with the survey), which entered a digital prototyping process. In addition to the collective or individual who proposed the selected ideas, a call for collaborators was opened. The goal was to engage people from diverse backgrounds and contexts in the process to ensure that the prototypes were more plural and inclusive. The open call application form, the evaluation criteria and the call for collaborators survey are available in S4–S6 Tables.

The prototyping process consisted of three half-days (4 hours for each session) in which citizen scientists devised, designed, and prototyped the proposals. The entire prototyping process was carried out in collaboration with the network of Ateneus de Fabricació de Barcelona [20], public digital fabrication facilities in Barcelona. First, the design session had the aim of designing the prototypes. The knowledge, experiences, and know-how of the participants (divided into two groups, one per prototype) were first mapped, and the tasks, activities, contents, people in charge, times and materials necessary to materialise the ideas were defined, considering the restrictions of time, budget and available tools. The required materials were purchased and collected during the next days. One week later, the prototyping session took place with the aim of prototyping the devised projects. Digital media such as hand tools and digital fabrication tools such as 3D printing, CNC, Laser Cutting, and Vinyl Cutters were used. To facilitate in-depth discussions and organise the work effectively, three canvas (knowledge mapping, prototype ideation, and prototype planning) were designed and used allowing the team to organise different elements such as the available human resources, the team knowledge, the tasks, and materials required, and the contents to develop. The templates of the canvas can be found in S7 Fig.

### Phase IV: Communication

The documentation of facts and learnings was considered during the whole project. The aim is that the information can circulate and be useful in other contexts under the principle of free culture so that it can be copied, distributed, modified, and improved. One action carried out for this purpose was the video documentation of the process. All project materials, including the video, are freely available on the project’s official website. Written consent from participants to appear in videos and photographs was obtained through an informed consent form.

Once the project finished, a session with participants was organised to discuss all the results of the process and co-elaborate the communication plan to disseminate them. To dynamise the co-creation of the communication plan, a participatory canvas was designed and used, which can be consulted in S8 Fig. The implementation of the different actions was equally distributed among the participants based on their previous experience.

### Evaluation

An online survey was created to determine if the process provided citizen scientists with new knowledge on the addressed issues and whether the results of the process were useful in the territory. Therefore, the survey was shared with those participants who attended more than one session of the process, excluding those entities or individuals who had a one-off collaboration. The survey questions can be found in S7 Table.

## Results

### Phase I: Participatory diagnosis

We obtained a total of 91 responses in the participatory diagnosis, 61 responses via the online survey, 17 responses in the kick-off meeting (participatory canvas), and 13 responses in the neighbourhood meeting (participatory canvas). At the city level (both in the online survey and kick-off meeting), most responders were between 31 and 50 years old and were living in Sant-Martí, Sants-Montjuïc and Eixample districts. On the other hand, at the neighbourhood level, the responders were all from the Sant Andreu district—where the selected neighbourhood, Trinitat Vella, is located—and mostly between 51 and 70 years old (Table 2 - Q1 and Q2). Note that, at the city level, all participants share their age (n = 78), but not all of them shared the neighbourhood where they were living (n = 75). In the question *“What aspects of the city do you think most affect your health*?*”*, air quality was the most voted in both cases, followed by noise in second place, and mobility and lack of natural areas with similar ratings in third place. We found differences in the results regarding noise, loss of biodiversity and temperatures, with noise being more relevant at the city level and biodiversity and temperatures more relevant at the neighbourhood level (Table 2 - Q3). In relation to the last question, “*Which group do you think is most affected by the planning and design of our cities*?*”*, participants identified the elderly as the most vulnerable group. In this question, there were also differences between the neighbourhood and the city: at the neighbourhood level, people with health problems and functional diversity emerged as more relevant while, at the city level, participants pointed to children and adolescents (Table 2 - Q4). Questions Q3 and Q4 allowed more than one answer. During the discussion with participants in the neighbourhood, participants pointed out that *“the neighbourhood used to be very close to a natural area with orchards some years ago*, *which were replaced by roads and an artificial park”* and related this as something negative that resulted in the loss of biodiversity in the neighbourhood.

**Table 2:** Complete reference of answers to the LAB CSU online survey.

Question	City *(78 participants)*		Neighbourhood *(13 participants)*	
	n	%	n	%
**Q1. How old are you?**				
Less than 18 years old	0	0,00%	0	0,00%
18–30	11	17,07%	3	23,07%
31–50	43	53,65%	3	23,07%
51–70	20	24,39%	7	53,84%
71–90	4	4,87%	0	0,00%
More than 90 years old	0	0,00%	0	0,00%
*Total*	*78*	*100*,*00%*	*13*	*100*,*00%*
**Q2. What neighbourhood do you live in?**				
Sant-Martí	18	24,00%	0	0,00%
Sants-Montjuïc	16	21,33%	0	0,00%
L’Eixample	15	20,00%	0	0,00%
Ciutat Vella	6	8,00%	0	0,00%
Sant Andreu	4	5,33%	13	100,00%
Sarrià-Sant Gervasi	3	4,00%	0	0,00%
Les Corts	3	4,00%	0	0,00%
Horta-Guinardó	2	2,67%	0	0,00%
Nou Barris	1	1,33%	0	0,00%
Gràcia	7	9,33%	0	0,00%
*Total*	75*	*100*,*00%*	*13*	*100*,*00%*
*Three participants did not answer this question.				
**Q3. What aspects of the city do you think most affect your health?** * multiple answers				
Air quality	61	28,37%	12	20,00%
Noise	49	22,79%	9	15,00%
Lack of natural areas	36	16,74%	9	15,00%
Mobility model	34	15,81%	9	15,00%
Lack of biodiversity	10	4,65%	7	12,00%
High temperatures	10	4,65%	6	9,00%
Lack of infrastructure for play and sport	6	2,79%	4	7,00%
Lack of space for socialising and caring	9	4,19%	4	7,00%
*Total*	*215*	*100*,*00%*	*60*	*100*,*00%*
**Q4. Which group do you think is most affected by the planning and design of our cities?** * multiple answers				
Elderly	45	21,53%	11	20,75%
Girls, boys and teenagers	44	21,05%	6	11,32%
People with health problems	29	13,88%	8	15,09%
People with disabilities	32	15,31%	8	15,09%
Migrants	7	3,35%	4	7,55%
Women	19	9,09%	5	9,43%
People with socioeconomic vulnerability	30	14,35%	9	16,98%
LGTBIQA+ collective	0	0,00%	1	1,89%
People working in public spaces	3	1,44%	1	1,89%
*Total*	*209*	*100*,*00%*	*53*	*100*,*00%*

The online version can be visited at this link.

In addition to these common questions, a specific question in the online survey *(Q5 = Considering these (or other) urban problems and their impact on health*, *which topics do you find more interesting to research and explore at the citizen level*?) allowed us to collect proposals for topics that people would be interested in investigating. Based on 59 proposals received, a total of 15 categories were created to regroup them, according to the keywords included in the answers. Most of the proposals were included in more than one category because they addressed different elements of the list. Many of them address mobility issues (n = 12), noise (n = 12), the use of public space (n = 15), natural areas (n = 14), air pollution (n = 11) or mental and physical health (n = 10). Less common categories were the need for space to socialise (n = 8), social inequalities (n = 6), consumption (n = 4), housing (n = 3), climate shelters (n = 3), urbanism with a gender perspective (n = 2), cohabitation and safety (n = 2), decision making (n = 2), and the impact of tourism (n = 1). As examples of the proposals received, one respondent suggested investigating *“the type of particles contained in the city’s air and how*, *as citizens*, *we can contribute to reducing them”* or *“how green spaces affect physical and mental health and how we as citizens can help to have more green spaces and what plants are the best to have in our city”*, and another participant suggested to investigate about *“how the current model of cities contributes to social*, *health and economic inequalities”*. A last question to find out if the respondents would be interested in getting involved in the project was also included *(Q6 = Do you want to get involved in a community project to work on these issues and contribute to the future of your city*?*)* and 44 respondents answered yes (53,7%), also giving their email addresses for further contact.

**Fig 2:**
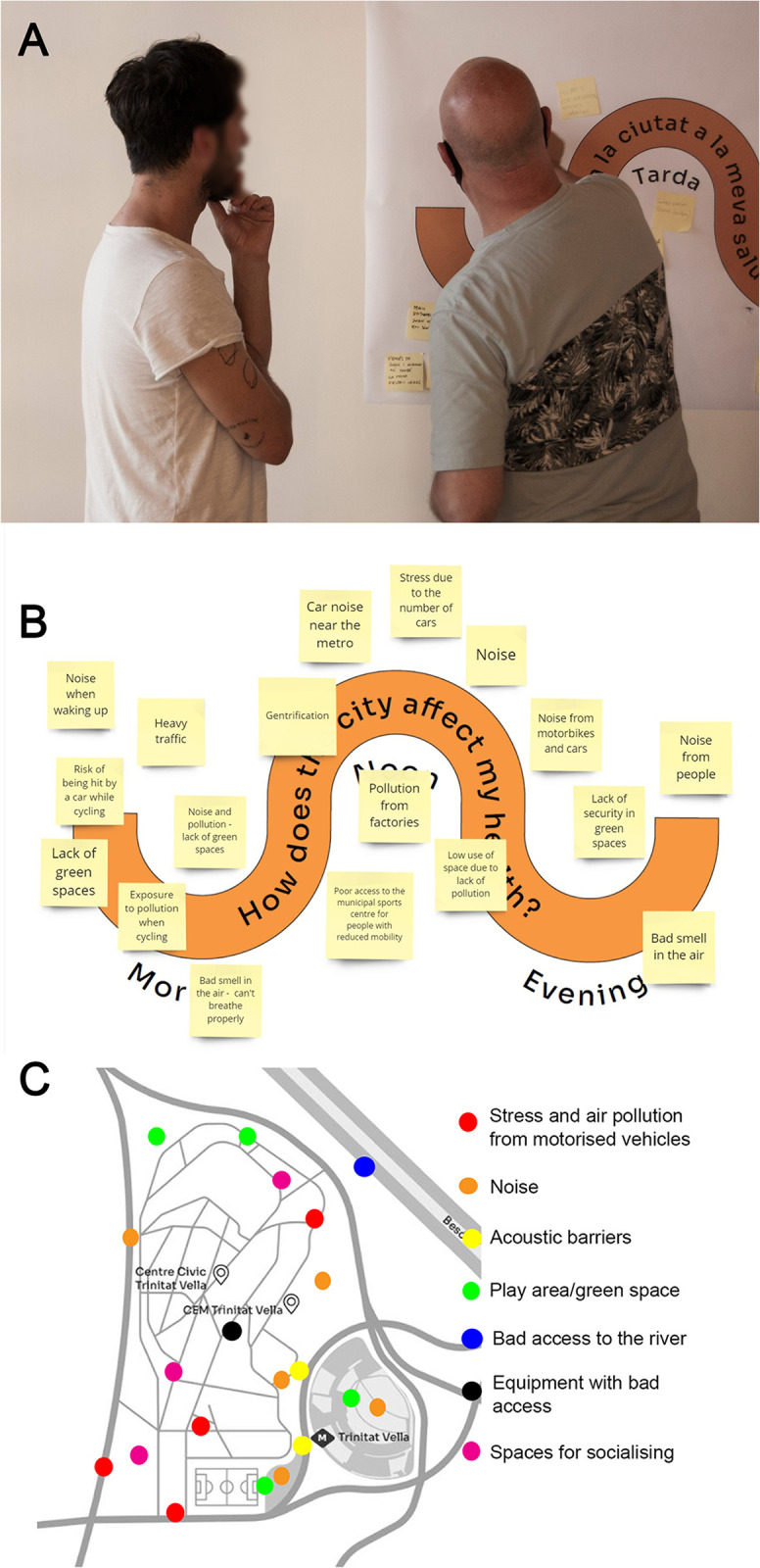
The process and results of participatory diagnosis. Two participants discussing in front of the Participatory Canvas to reflect on the impact of urban planning on people (A), compilation of the ideas reflected by all participants during the session (translated from the original Spanish version) (B), and a compilation of relevant places of interest in the neighborhood proposed by participants in relation to urban planning and (translated from the original Spanish version).

Finally, we did a collective mapping of ideas and relevant elements related to urbanism and health in the neighbourhood (Fig 2A). The most representative ideas that emerged were the unpleasant odours, the noise caused by cars, motorbikes and people, and the stress caused by the traffic (Fig 2B). In relation to the mapping of elements, both positive and negative elements were identified, such as the presence of green and socialising spaces, areas where noise is perceived, areas with poor access or areas where there is stressful exposure to traffic (Fig 2C). Some participants remarked on the high number of cars that pass around the neighbourhood every day and how this "*generates stressful situations*, *such as elderly people having to cross the road quickly*, *something that has led to some falls*". They also mentioned the continuous noise generated by cars and the bad smell in the air, due to both factories and traffic.

### Phase II: Citizen science

#### II.I Definition of the research question and hypothesis

To define the research question, three assumptions were agreed upon with participants. The question should be (1) based on the findings of the participatory diagnosis, (2) answered by taking data in the short term (within the framework of the project) and (3) have a local and concrete scope. Regarding the first requirement, participants prioritised focusing on those aspects that were most representative: air quality, noise and temperature in the neighbourhood. For the second and third requirements, participants agreed to collect quantitative data for two weeks that could be obtained from the neighbourhood. Upon this, different questions were raised, based on the more relevant topics: *Is the Plaça de la Trinitat Vella square a healthy space*? *Is Trinitat Vella Park a climate shelter*? *Is Trinitat Vella Park a healthy space*? *At what level of air pollution am I exposed to as a resident of Trinitat Vella*? and *Which is the most and least polluted place in the neighbourhood*? The term "healthy" and the characteristics of a climate shelter were discussed with the participants. Finally, they decided to choose the research question: *At what level of air pollution am I exposed to as a resident of Trinitat Vella*? because of its concreteness and the ease with which it can be answered in the chosen terms.

#### II.II Definition of the data collection protocol and process

Among the proposed tools, they chose to measure NO_2_ with Palmes diffusion tubes (passive air samplers), and noise and suspended particles using Smart Citizen Kits (SCKs) and selected different places in the neighbourhood to install the tubes and the SCKs. The Palmes diffusion tubes were chosen for their reliability, simple (un)installation and ease of obtaining results. The SCKs were chosen because of the range of variables they measure (temperature, particulate matter, noise, etc.) and the ease of (un)installation. In relation to the diffusion tubes, the participants selected the measurement sites using the map from the participatory diagnosis as a basis, and a group was agreed upon for installation the following week. In addition to the tubes installed outdoors in the public space, two participants wore a personal exposure tube during the whole day, both indoors and outdoors, for 7 days. One of the participants collected all the tubes after the exposure monitoring and sent them to the Gradko Environmental lab for analysis. Regarding the SCKs, three participants volunteered to place the sensor on their windows/balconies to monitor outdoor air quality.

#### II.III Data analysis

NO_2_ concentrations (in μg/m^3^) were adjusted for temporal variation to remove the seasonal meteorological influence and make the measurements comparable to each other. Briefly, daily NO_2_ data was obtained for the entire year of the study (2022) from a background reference station nearby (Vall Hebrón, Barcelona). Daily ratios between daily values and the annual averaged NO_2_ were calculated and applied to each measurement period. SCK data was recorded every minute on a micro-SD card and after the monitoring period, it was downloaded. We analysed hourly means for noise and PM2.5 (suspended particles that are 2.5 μm or less in diameter).

#### II.IV Results

The obtained results in the neighborhood have been interpreted in relation to three reference values, which are outlined below. On one hand, the new World Health Organization (WHO) guidelines that consider the latest body of evidence on the health impacts of different air pollutants and aim to favor evidence-based legislation and policies to improve air quality, establishes a limit of 10 μg/m3—annual mean of ambient NO_2_ and 5 μg/m3—annual mean of PM 2.5. On the other hand, the European Union (EU) proposes an extensive body of legislation which establishes standards and objectives for several pollutants in air in the member states and establishes a limit of 40 μg/m3—annual mean of ambient NO_2_ and 25 μg/m^3^—annual mean of PM 2.5. The limits established by the EU are, so far, more flexible than those recommended by the WHO. However, it is worth mentioning that the EU has recently proposed an update to its limits based on the WHO guidelines (still pending approval) to 20 μg/m^3^—annual mean of ambient NO_2_ and 10 μg/m^3^—annual mean of PM 2.5 [58].

**Fig 3:**
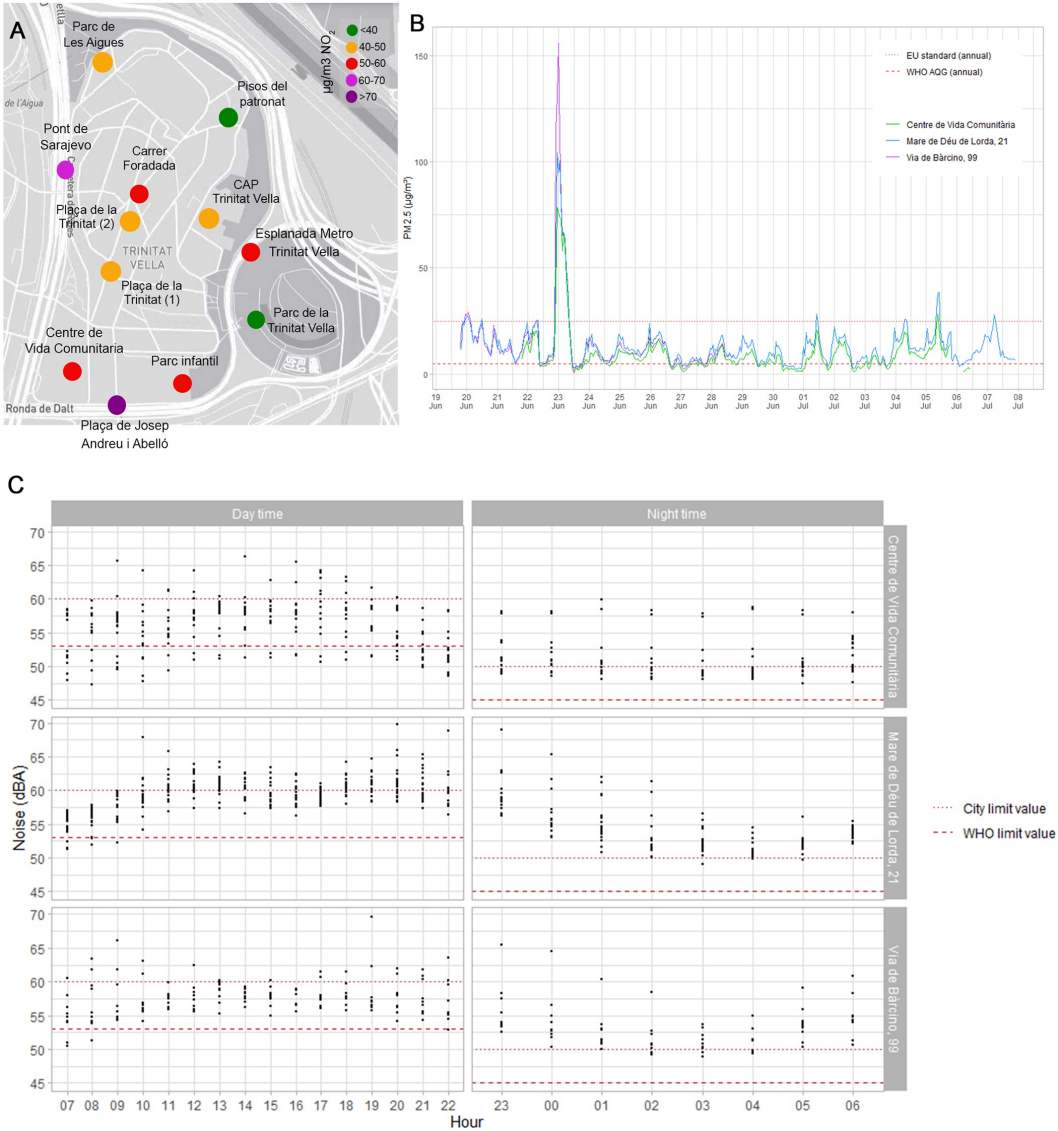
Results of air quality and noise in the Trinitat Vella neighborhood. Ambient NO_2_ concentration in μg/m^3^ during the exposure period at different points in the neighborhood, represented with a color range according to concentration (see legend in the figure) (A), PM 2.5 particulate matter concentration (y-axis) at three different locations in the neighborhood (see figure legend) over the exposure period (x-axis) (B), and detected noise levels (dBA) (y-axis) at three different locations in the neighborhood (see identification in each graph) over the exposure period (x-axis) during both day and night (C).

Out of the 12 fixed monitored locations, 10 showed ambient NO_2_ concentrations above the limit values established by the EU (40 μg/m^3^—annual mean). In relation to the limits recommended by the WHO air quality guidelines, all the points exceeded the maximum recommended levels (10 μg/m^3—^annual mean). The most polluted location in the neighbourhood was Josep Andreu i Abelló square, with a concentration of 75,72 μg/m^3^, and the least polluted site was the area around the patronage flats (*Pisos del patronat*), with a concentration of 37,25 μg/m^3^ (Fig 3A). All the measurements will be beyond the limit values by the time the new EU air quality standards are approved (20 μg/m^3^—annual mean). As a reference to the potential impact of an increase in NO_2_ concentrations on neighbours, an annual increase of 10 μg/m3 of NO_2_ contributes to a 4% increase in mortality [59].

Regarding particulate matter concentrations, the three measurement locations showed a consistent trend over the days, indicating a similar PM 2.5 exposure at different locations in the neighbourhood. In relation to the limits, all locations showed concentrations mainly above the threshold recommended by the WHO (5 μg/m^3^—annual mean) and below the legal limits set by the EU (25 μg/m^3^—annual mean) (Fig 3B). However, most of these concentrations will be beyond the limit values by the time the new EU air quality guidelines are approved (10 μg/m^3^—annual mean) [22]. The peak in PM2.5 levels observed on June 23rd was attributed to the bonfires and fireworks that were set off during the Saint John’s Eve celebration.

Regarding noise exposure, differences can be seen between the different measurement sites. Throughout the day, *Mare de Déu de Lorda* is the location with the highest noise levels, showing a high proportion of measurements exceeding the legal limits established in the municipal ordinance for residential areas (60 dB(A)) (Fig 3C—daytime). The other two locations showed, in general, noise levels above the WHO recommendations (53 dB(A)). At night, all three measurement points mostly showed levels above the EU legal limits (50dB(A)) and WHO recommendations (45dB(A)) (Fig 3C—nighttime).

### Phase III: Social innovation

**Fig 4:**
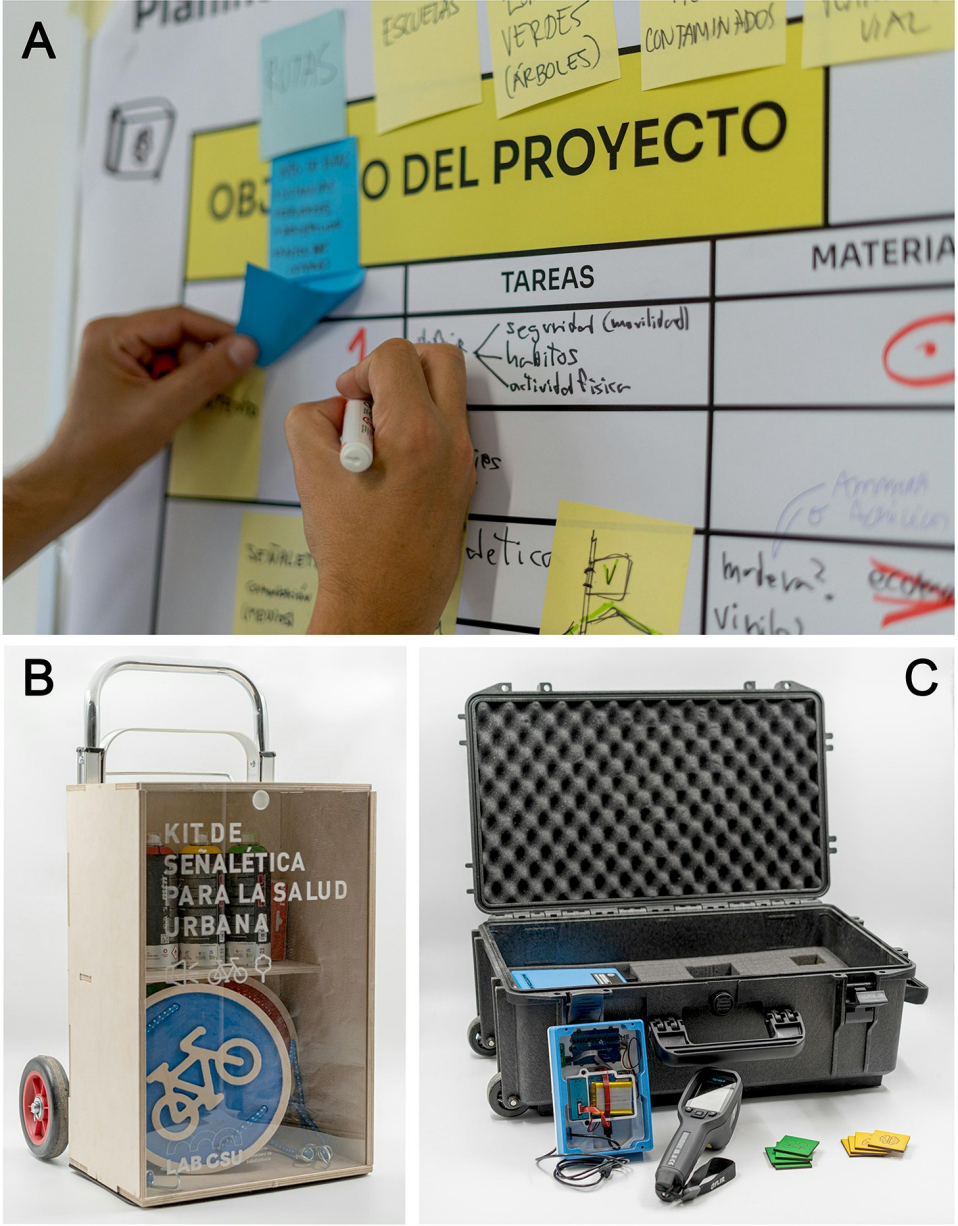
The process and results of social innovation. Image of a participant jotting down ideas on the Work Organization Canvas (A), the Urban Signaling Kit (B), and the Environmental Suitcase (C).

The open call received a total of 26 proposals, the majority coming from residents of the Trinitat Vella neighbourhood (55,2%), followed by entities and organisations (13,8%) and other citizens from Barcelona (31%). All the proposals were framed within the categories of community networks (31%), inclusion and social integration (13,8%), physical and mental health (3,4%), air pollution (20,7%), local biodiversity (13,8%) and urban mobility (17,2%). A complete list of the proposals received can be consulted in S7 Table. According to the pre-established criteria, the jury chose two proposals: *Sa i actiu al meu barri* (number 4: Healthy and active in my neighbourhood) and the Environmental educational suitcase (number 11), based on the criteria specified in the methods section. Then, a call for collaborators was open and new citizens joined the prototyping process, working collaboratively for two days to develop the projects (Fig 4A). The final outcomes were: (1) an urban signalling kit that seeks to dynamise interventions in public spaces to raise awareness about the health impacts of the lack of green spaces, noise and air pollution (Fig 4B); and (2) an educational environmental suitcase, which seeks to raise students’ awareness on the impact of urban planning on health and to give continuity to the CS process in the neighbourhood through collaboration with institutes (Fig 4C).

### Phase IV: Communication

Among the elements to be disseminated, participants co-identified air and noise pollution data resulting from the CS process, the prototypes resulting from the social innovation process, the project’s methodological framework and the video documentary. Concerning the target audience, children, teenagers, neighbours and the public, administrations and the scientific community were identified. Specific actions were proposed for each element and target audience, e.g., training members of local facilities to follow up the use of prototypes with children and adolescents, giving interviews on local radio or television to reach the public, or writing the present article to reach the scientific community. All the ideas agreed upon during the session are listed in S8 Table. Of the actions identified, at the time of writing this article, some have already been implemented, including interviews on local radio and television and an informative walk through the neighbourhood. More information about these activities can be found on the official webpage of the project.

### Evaluation

A large proportion of participants that answered the survey had participated in all (31,57%) or most (26,31%) phases of the project (S9 Table—Q1). More than half of the participants (52,6%) reported learning a lot about urban planning and health during the project (S9 Table—Q2), especially about air pollution (36,11%) and noise (30,55%), and less about blue areas (11,11%) (S9 Table—Q3). A high percentage (42,10%) of the participants responded that they had changed their own perception of how urban planning influences health (S9 Table—Q4). Most participants (84,21%) have acquired new knowledge about how research is organised (S9 Table—Q5) and all participants reported having learned about new tools for environmental data collection, up to four (26,31%) or more (36,84%) (S9 Table—Q6). Finally, participants were asked whether they considered that the results of the project would be useful in the territory. Most of the answers were positive, highlighting the need to include the neighbours and the administrations to achieve future improvements in the territory (S9 Table—Q7).

## Discussion

This paper describes the first pilot of the CSU LAB, a project funded by the Barcelona City Council through the Fundació Bit Habitat, which aims to promote a space for participation and research on urban health by combining CS and social innovation. The project has facilitated a horizontal and participatory research process through the development of a participatory diagnosis, co-design of a research question, co-organisation of the work and data collection, and collaborative analysis and interpretation of the results. Based on the conclusions obtained, a process of social innovation was promoted, where people from different profiles and backgrounds participated in the proposal, design, and prototyping of solutions for the problems detected in the neighbourhood. Finally, a communication plan was jointly designed to disseminate both the results and prototypes resulting from the process. The project had the core involvement of Trinitat Uneix, a neighbourhood association in Trinitat Vella. Its members participated in all phases of the project, linking the process to the territory by communicating with other stakeholders and facilities in the neighbourhood, and co-designing the communication plan. For instance, they linked the prototypes generated with local facilities in the neighbourhood and communicated the results of CS in different political decision-making spaces.

The participatory diagnosis enabled the analysis of the elements of urban planning that participants perceived as relevant. Air pollution and noise were identified as relevant topics both at the level of Barcelona (city) and Trinitat Vella (neighbourhood), indicating their potential relevance and current concern in other neighbourhoods of the city as well, consistent with a previous study [23] However, certain elements, such as the loss of biodiversity, noise, or rising temperatures, became more important in the neighbourhood. The orography of the neighbourhood and its direct exposure to noise (surrounded by several main roads), along with climate change vulnerability mapping [60] and the direct testimonies of neighbours helped to understand the greater concern for these issues. When assessing possible vulnerable populations affected by urban planning in Trinitat Vella, there was a greater concern towards socio-economic vulnerability, as it is one of the neighbourhoods with the lowest per capita income in Barcelona. For example, one of the participants—a person with functional diversity—explained how urban planning hinders his day-to-day life in the neighbourhood, which helped to discuss urbanism also from this perspective. The inclusion of these different perspectives that may not typically be included in participatory research (people with functional diversity or economic vulnerability) is crucial to ensure the democratizing capacity of participatory projects [61].

The analysis of the air quality and noise data obtained in the neighbourhood revealed levels that exceed the standards recommended by the WHO [62] and, very often, the limits set by the EU [63]. This situation is shared, to a greater or lesser extent, by other neighbourhoods in the city [64]. Note that this data will need to be rescaled once the EU approves its proposal to set new air quality standards [58] (annual average of 5 μg/m^3^ for PM2.5 and 10 μg/m^3^ for NO_2_). Surprisingly, this first approach to a descriptive investigation of air quality in the neighbourhood showed levels above those reported by the Barcelona Public Health Agency through its official network of monitoring stations [65]. The difference in NO_2_ and PM2.5 concentrations could be explained by methodological differences, such as the location, the monitoring period and length (a few days/weeks mean in this project versus the annual mean in the case of the official monitoring stations). However, this situation could suggest that this research have offered a more specific resolution of air quality at the neighbourhood level, while also providing complementary information to the official measurement stations, which provide a broader city-wide or district-wide resolution scale. Although the quality of data obtained through CS continues to be questioned in some contexts [42], a recently published CS study on air quality confirm that street-level spatial resolution provides exposure measurements that do not necessarily match those from monitoring stations [66] In a broad sense, the co-creation of research through a participatory process entails that the data furnishes an air quality assessment of regions that hold significance for residents. This empowers them to guide the trajectory of research according to societal requirements and contribute insights that might not have garnered sufficient recognition within the scientific establishment [61].

In relation to the social innovation stage, the collaboration of the *Ateneus de Fabricació Digital* was key to articulating and linking the process to the territory. It also offered an optimal space for its replicability, both locally and in other contexts where similar structures may exist. One of the main challenges that was faced was to reach out to the community and encourage them to present ideas to the open call. On the one hand, the dissemination of the online survey helped us to capture more complex ideas, since this tool provided the possibility of writing and attaching images or diagrams. However, the use of online surveys could exclude older or less digitally literate citizens from participation, so the organisation of the face-to-face event during Park(ing)Day helped us to reach out, in a more informal way, to a diversity of people in the neighbourhood. The design and prototyping sessions were cooperative and inclusive, with the participation of a geographically (from different neighbourhoods of the city), professionally (with different work experience or studies), age and gender diversity of actors. Despite that, organising time and work during a short and intense prototyping process could have been challenging so, to overcome this difficulty, a support team was invited to the sessions (one designer and one digital prototyping expert), which helped to organise the ideas, time, and work efficiently to reach the results proposed by each working group. In summary, the most relevant aspect of social innovation is not the result but the process, valuing the contributions of all participants and demonstrating how technology, used for the right purpose, can empower citizens to materialise their ideas and generate a positive impact in their neighbourhoods.

Within the scope of the project, one of the evident constraints has been time limitations. In its initial pilot phase of one year, the project allocated a 4-month timeframe for CS activities. This time constraint has occasionally posed challenges, given the participants’ restricted availability and their diverse array of commitments and restrictions. Furthermore, this time limitations (and budget limitations) also exerted an influence on the project’s investigative focus. For example, the data collection process, which was estimated to span approximately two weeks (based on project time and budget constraints), imposed restrictions on the feasibility of conducting extended-duration measurements. Consequently, this narrower timeframe influenced the refinement of the research question—something already acknowledged in existing literature [23].

Another constraint arises from the demands in terms of time and resources required to involve diverse groups of citizens, which is where the true richness of the process resides. This diversity is important, both in terms of quantity and variety, including not only the more privileged or motivated citizens. It allows for the definition of a more inclusive research agenda, as well as contribute to providing solutions that address dimensions that are not typically addressed [67]. Despite concerted communication efforts by both project promoters and Trinitat Uneix, the engagement of unorganized citizens (those who do not belong to any group or organization) remained peripheral throughout the project. The modular nature of the framework facilitated citizen participation even if it was at a specific moment of the project, as this participation could be adapted to the citizens’ interests, necessities, and availability. However, most of the work was carried out by a small group of citizens, which is also referenced as a limitation in participatory processes [68, 69]. In this regard, some practices could be implemented to improve citizen engagement and contribution to the project, such us offering monetary and public online acknowledgement rewards [70].

One last limitation was that participants exhibit varying levels of resistance to the collective ideation, design, and prototyping process. In general, such methodologies, tools, and processes are not commonly encountered in our daily lives, leading to a slight aversion to change that can stall and delay the process. This generates stress and frustration as participants don’t have absolute control over the outcome and are required to trust unfamiliar individuals to achieve a common goal. Drawing from our experience, once that aversion threshold is overcome, everything flows smoothly and people enjoy and value the process, building community through their shared efforts.

The CSU LAB has been the first successful pilot to design and test the described methodology, which seeks to combine co-created CS and SI to empower citizens in their active involvement in research and innovation related to urban health. The methodology and its resources, which are open access, could be valuable tools for other facilities and institutions aiming to promote or support the urban transformation processes taking place in their territories [17]. Although this first pilot project focused (by decision of the research community) on air quality and noise, the CSU LAB methodology allows addressing other issues related to urban health, including but not limited to natural areas, urban design, sustainable mobility, and rising temperatures. It is therefore a versatile methodology that allows current and future urban health challenges to be addressed in a participatory, interdisciplinary, collaborative, and horizontal manner. This provides scholars, managers, and policymakers with a framework that allows for the collection of scientific evidence on a complementary scale that allows a more comprehensive and cross-cutting diagnosis of various urban health issues, and aids in the design and implementation of urban infrastructures and interventions based on empirical and participatory evidence. This provides scholars, managers, and policymakers with a framework that allows for the collection of scientific evidence on a complementary scale that allows a more comprehensive and cross-cutting diagnosis of various urban health issues, and aids in the design and implementation of urban infrastructures and interventions based on empirical and participatory evidence.

### Conclusions

This paper describes a methodological framework to promote participatory processes with the aim of empowering communities in addressing urban health challenges. The methodology, which combinesCS and SI, allowed the community that participated in the first pilot to co-design and develop a diagnosis and research on air quality and noise in the neighbourhood and to collaborate in the design and implementation of two prototypes to raise awareness of this issues in the territory and to involve students from the neighbourhood in the research process in the medium term. Moreover, the project has also provided the community with a space to discover and discuss the existing evidence of the impact of the urban environment on health, has raised community awareness on this topic and generated results for advocacy of a healthier neighbourhood model. The framework described can be used by public facilities, administrations, entities, and organizations to address the urban transformation processes in a participatory, interdisciplinary and collaborative way.

## Supporting information

S1 Table List of actors involved in the meetings to select the pilot neighbourhood to implement the project. (DOCX)

S2 Table List of questions in the LAB CSU online survey. (DOCX)

S3 Table List of locations and days when passive diffusion tubes and Smart Citizen Kits were installed. (DOCX)

S4 Table Open call application form. (DOCX)

S5 Table Open call evaluation criteria. (DOCX)

S6 Table Call for collaborators survey. (DOCX)

S7 Table List of proposals received in the open call for ideas. (DOCX)

S8 Table Co-created communication plan. (DOCX)

S9 Table Evaluation online survey. (DOCX)

S1 Fig Participatory diagnosis canvas. (DOCX)

S2 Fig Canvas to reflect on the impact of urban planning on human health. (DOCX)

S3 Fig Canvas to reflect on the individual’s impact on the city. (DOCX)

S4 Fig Problem definition canvas. (DOCX)

S5 Fig Hypothesis and research question definition canvas. (DOCX)

S6 Fig Work organization canvas. (DOCX)

S7 Fig Knowledge map canvas. (DOCX)

S8 Fig Prototype ideation canvas. (DOCX)

S9 Fig Prototype planning canvas. (DOCX)

S10 Fig Communication plan design canvas. (DOCX)

## List of abbreviations

CSU LAB: Citizen Urban Health Laboratory
WHO: World Health Organization
EU: European Union
SCKs: Smart Citizen Kits
PM: Particulate Matter
CS: Citizen Science
SI: Social Innovation
