# Supplementary material for: Citizen science and social innovation as citizen empowerment tools to address urban health challenges: The case of the urban health citizen laboratory in Barcelona, Spain
Source: PLoS One. 2024 Mar 13;19(3):e0298749. doi: 10.1371/journal.pone.0298749 (PMC10936789; doi:10.1371/journal.pone.0298749)
Supplement: S1 Table — (DOCX) [file pone.0298749.s001.docx]

**Table S1. List of actors involved in the meetings to select the pilot neighbourhood to implement the project.**

| **City of Barcelona** | | **Districts** | **Trinitat Vella neighbourhood** |
| --- | --- | --- | --- |
| **Step 1** | **Step 2** | **Step 3** | **Step 4** |
| Ecología, urbanismo, infraestructuras y movilidad \| Participación y Comunicación | Departament d'Associacionisme i Iniciatives ciutadanes \| Direcció d'Acció Comunitària | Aula ambiental  de Sant Andreu | Trinitat Uneix |
| Pla comunitari del Besòs |  | Pla comunitari de Verdum | Esplai La Tortuga |
| Consorci Besòs |  | Pla comunitari de Poble Sec | Amigues de l'Olivera |
| Regidoria Sant Martí |  | Pla Comunitari del Besòs | Grup d’escolta activa |
| Ecología, urbanismo, infraestructuras y movilidad \| Aules Ambientals i Escoles + Sostenibles |  | Cap de Projecte del Pla de Barris, La Trinitat Vella, el Bon Pastor i Baró de Viver |  |
