# Supplementary material for: Citizen science and social innovation as citizen empowerment tools to address urban health challenges: The case of the urban health citizen laboratory in Barcelona, Spain
Source: PLoS One. 2024 Mar 13;19(3):e0298749. doi: 10.1371/journal.pone.0298749 (PMC10936789; doi:10.1371/journal.pone.0298749)
Supplement: S2 Table — (DOCX) [file pone.0298749.s002.docx]

**Table S2. List of questions in the LAB CSU online survey** (English translation from the original version, which can be visited at this [link](https://form.typeform.com/to/FOeyW4tz?typeform-source=www.google.com)).

**Introduction**

LAB CSU is a space that promotes the development of creative and collective ideas around urban health through experimentation and collaborative learning. With your help, we will build a first-citizen diagnosis of urban health.

| **Questions** | **Answer options** |
| --- | --- |
| How old are you? | Less than 18 years old  18-30  31-50  51-70  71-90  More than 90 |
| In which neighbourhood do you live? | Open question |
| Which aspects of the city do you think affect your health the most? This question is required  **You can choose up to 3* | Air quality  Noise  The lack of access to green and blue spaces (sea, rivers, etc.)  The lack of public infrastructure for physical activity and play  The lack of spaces for socialisation and care  The urban mobility model  Low capacity of public space to protect from temperature (high and low)  Loss of biodiversity  Other |
| Which group do you think is most affected by the planning and design of our cities?  **You can choose up to 3* | Women  Elderly  Children and adolescents  People with health problems  People with mobility problems  Migrants  Low-income populations  LGTBIQA+  People working in the public space  Other |
| Considering these (or other) urban problems and their impact on health, which topics do you find more interesting to research and explore at the citizen level? | Open question |
| Do you want to get involved in a community project to work on these issues and contribute to the future of your city? | Yes  No |
| Leave us your email to receive more information | Open question |
