# Supplementary material for: Citizen science and social innovation as citizen empowerment tools to address urban health challenges: The case of the urban health citizen laboratory in Barcelona, Spain
Source: PLoS One. 2024 Mar 13;19(3):e0298749. doi: 10.1371/journal.pone.0298749 (PMC10936789; doi:10.1371/journal.pone.0298749)
Supplement: S3 Table — (DOCX) [file pone.0298749.s003.docx]

**Table S3. List of locations and days when passive diffusion tubes and Smart Citizen Kits were installed.**

| **Parameter** | **Tool** | **Location** | **Address** | **Days** |
| --- | --- | --- | --- | --- |
| NO_2_ | Palmes’ diffusion tubes | Pont de Sarajevo | Carrer d'Almassora, 732, 08033 Barcelona | 7 |
|  |  | Plaça de Josep Andreu i Abelló | Josep Andreu i Abelló, 161, 08033 Barcelona | 7 |
|  |  | Plaça de la Trinitat (1) | Plaça de la Trinitat, 14, 08033 Barcelona | 7 |
|  |  | Plaça de la Trinitat (2) | Plaça de la Trinitat, 1, 08033 Barcelona | 7 |
|  |  | Pisos del patronat | Via de Bàrcino, 27-65, 08033 Barcelona | 7 |
|  |  | Parc infantil | Carrer de la Mare de Déu de Lorda, 34, 08033 Barcelona | 7 |
|  |  | Parc de Les Aïgues | Carrer Torrente de la Perera, 107, 08033 Barcelona | 7 |
|  |  | Parc de la Trinitat Vella | Pg. de Santa Coloma, 60, 08030 Barcelona | 7 |
|  |  | Esplanada Metro Trinitat Vella | B-20, 11-37, 08033 Barcelona | 7 |
|  |  | Centre de Vida Comunitària | Via Favència, 399 Ctra de Ribes amb, Via Favència, 08033 Barcelona | 16 |
|  |  | Carrer Foradada | Carrer de la Foradada, 36, 08033 Barcelona | 7 |
|  |  | CAP Trinitat Vella | Via de Bàrcino, 88, 08033 Barcelona | 7 |
|  |  | Personal exposure |  | 7 |
|  |  | Personal exposure |  | 7 |
| Particulate matter and Noise | Smart Citizen Kit | Centre de Vida Comunitària | Via Favència, 399 Ctra de Ribes amb, Via Favència, 08033 Barcelona | 14 |
|  |  | Participants’ Home | Via de Barcino, 99, 08033 Barcelona | 9 |
|  |  | Participants’ Home | Carrer de la Mare de Déu de Lorda, 21, 08033 Barcelona | 18 |
