# Supplementary material for: Citizen science and social innovation as citizen empowerment tools to address urban health challenges: The case of the urban health citizen laboratory in Barcelona, Spain
Source: PLoS One. 2024 Mar 13;19(3):e0298749. doi: 10.1371/journal.pone.0298749 (PMC10936789; doi:10.1371/journal.pone.0298749)
Supplement: S4 Table — (DOCX) [file pone.0298749.s004.docx]

**Table S4. Open call application form.**

**Introduction**

Do you have an idea that could help improve the quality of life in your neighbourhood?

Present a proposal to work on in a collaborative way.

*The call for proposals is open until 20 September 2022.

**More information and terms and conditions at https://www.labcsu.com/recursos/

| **Questions** | **Answer options** |
| --- | --- |
| Where do you live? | Open question |
| Idea: Describe briefly your proposal and its objective | Open question |
| What local problem does your proposal address? | Urban mobility  Local biodiversity  Air pollution  Community networks  Social inclusion  Gender perspective  Data and smart city  Other |
| Collaborators: Which people or groups do you think could contribute to your proposal? | Open question |
| Upload a drawing, sketch or photo of reference of your proposal (optional) | Open question |
| Define a name for your proposal | Open question |
| What is your name? | Open question |
| Leave us your email address so we can keep in touch | Open question |
