# Supplementary material for: Citizen science and social innovation as citizen empowerment tools to address urban health challenges: The case of the urban health citizen laboratory in Barcelona, Spain
Source: PLoS One. 2024 Mar 13;19(3):e0298749. doi: 10.1371/journal.pone.0298749 (PMC10936789; doi:10.1371/journal.pone.0298749)
Supplement: S5 Table — (DOCX) [file pone.0298749.s005.docx]

**Table S5. Open call evaluation criteria.**

TERMS AND CONDITIONS CALL FOR LAB COLLABORATORS CSU

This call is open to all those interested in collaborating on one of the selected citizen proposals:

- *Sa i actiu al meu barri*: Design and implement circuits, challenges, good practices and advice in different parts of the neighbourhood by means of playful signage on public roads to encourage physical activity and promote people's mental health.
- Environmental Suitcase: a didactic kit for the analysis of environmental pollution indicators to educate and to raise awareness in the knowledge and care of the environment among children and young people in schools.

**Important aspects**

- The two proposals are the starting point for collective work, but always under the principle that the ideas must be open, flexible, mutating and enriching in these processes of collective practice.
- The Laboratory will be implemented in the Trinitat Vella neighbourhood Budget €1,500 per prototype for each project.
- The call for proposals is open until 30 September.

**Who can participate?**

Anyone interested in any of the selected projects, regardless of their level of training, specialisation or experience. You don't need to live in Trinitat Vella, but the projects will be developed in this neighbourhood. The figure of the collaborator is fundamental in the approach and development of the LABCSU workshops, which are conceived as spaces for collaborative work and the exchange of knowledge and know-how. The workshops are based on a horizontal relationship between the mentors, the project promoters and the collaborators themselves. Collaborators can form part of the development team of one of the selected proposals, contributing their knowledge and ideas, while learning from the rest of the group and the workshop mentors.

# **Calendar and key dates**

LABCSU will be developed in the following phases:

- Call for collaborators: until the 30^th^ of September.
- Presentation of promoters and collaborators: the 6^th^ of October (18h).
- Face-to-face workshops: the 14^th^ of October (16h-19h) and the 15^th^ of October (9h-14h).
- Closing event and presentation of results: to be confirmed.

# **Registration**

You can register by filling in the following [registration form](https://form.typeform.com/to/pJR9Rkkt) or by sending an e-mail to hola@labcsu.com answering the following questions:

- What project would you like to collaborate on?
- What are your motivations and expectations of the project?
- What can you contribute as a collaborator? o What are your knowledge, skills or experience that you can bring to the project?
- Can you attend all Labcsu prototyping workshops? Which ones can't you attend?

**Production Workshop**

This call is open to all those interested in collaborating on one of the 2 proposals selected to be developed from the 6^th^ to the 15^th^ of October. We will have a team of experts in different areas who will be in charge of mentoring the workshop.

Due to the limited capacity, a maximum of 8 people per project will be selected. The idea is to generate the most heterogeneous groups possible, so the selection will take into account the origin, gender, profile and maximum time of attendance at the workshop. The selected collaborators will be announced on 30^th^ September.

The main purpose of the Citizen Laboratory is to create a community of learning and practice, working together to promote creative ideas that improve the living conditions of the Trinitat Vella neighbourhood.

Under a collaborative and interdisciplinary approach, LABCSU fosters conditions that enable the activation of collective listening, observation and intelligence among participants from different backgrounds linked to the same context, who have common interests, needs and desires.

In this space for meeting and cooperation, we work from the logics of experimentation and prototyping, prioritising the process over the result, for the collective generation of knowledge that subsequently circulates and can be useful in other contexts, under the principle of free culture for copying, distributing, modifying and improving.

# **Organisation’s commitments:**

LICHEN IS and ISGlobal, as promoters of LAB CSU, are committed to:

- Provide materials for the development of each of the projects according to the needs and capacities of the organisation.
- Providing, as far as possible, the necessary means to carry out the selected projects, including materials and equipment. Access to and use of the technical means provided is coordinated and supervised by the organisation. The costs of material and technical equipment that have not been requested or approved sufficiently in advance cannot be guaranteed.
- Accompanying participants in documenting the projects and publishing the results, promoting free culture and the dissemination of the knowledge produced during the process.
- Provide conceptual, technical and methodological advice on mentoring and mediation.
- To make a digital platform available to the working groups as an open repository for the projects, documentation and resulting prototypes.
- Dissemination of the projects through all the project's communication channels, media and social networks, and through other collaborating entities.
- Facilitating the intensive weekends of the workshops.
- Maintain a respectful attitude towards all people. Expressions of hatred or intolerance related to gender, race, ethnicity, diversity, social status, sexuality, religion or origin will not be accepted.

# **Participants' commitments:**

- Attending the workshops on the scheduled dates and other complementary activities that may contribute to the production processes in the teams involved.
- Be available to communicate with the organisation during the planned dates.
- Document the projects through the means offered by the organisation and provide the resulting materials.
- Accept and encourage the participation of people interested in collaborating. Recognise and accredit the participation and contribution of each member of the team.
- Take care of the materials, the space and the work environment during the development of the workshops.
- To publicly present the projects at the final closing event. As well as subsequently providing materials and/or prototypes developed for public exhibition.
- Promote free culture and disseminate the knowledge developed (methodologies, tools, texts) so that it is available under open licences.
- Maintain a respectful attitude towards all people. Expressions of hatred or intolerance related to gender, race, ethnicity, diversity, social status, sexuality, religion or origin will not be accepted.

# **Limitations of Responsibility**

The organisation is not responsible for the use of data or contents that may be used by the participant. Likewise, the organisation is not responsible for the copyright or any other rights that correspond to third parties in accordance with the respective laws.

# **Interpretation and modification of the terms of this call for applications**

# The organisation may make any modifications and interpretations it deems appropriate, in order to clarify the cases established. All circumstances not foreseen in this call will be resolved by the organisation. The decisions, the qualification and the result of the selection of projects are final. Participation in this call for applications implies acceptance of all the terms and conditions of the competition.
