# Supplementary material for: Citizen science and social innovation as citizen empowerment tools to address urban health challenges: The case of the urban health citizen laboratory in Barcelona, Spain
Source: PLoS One. 2024 Mar 13;19(3):e0298749. doi: 10.1371/journal.pone.0298749 (PMC10936789; doi:10.1371/journal.pone.0298749)
Supplement: S6 Table — (DOCX) [file pone.0298749.s006.docx]

**Table S6. Call for collaborators survey.**

**Introduction**

Production and Prototyping Stage: Would you like to participate in the Citizen Lab by collaboratively developing one of the selected proposals?

*The call is open until 1 October 2022.

**More information and terms and conditions at https://www.labcsu.com/recursos/

The ideas selected are as follows:

01- Environmental Educational Suitcase: didactic and playful kit to monitor pollution on a local scale and raise awareness of environmental care among children and young people in Trinitat Vella.

02- *Sa i actiu al meu barri*: circuits, challenges, good practices and advice in different locations of the neighbourhood to encourage physical activity and improve people's mental health.

Important information: In the next stage of production, these initial ideas are transformed and enriched with the contributions of a diverse group of collaborators.

| **Questions** | **Answer options** |
| --- | --- |
| What project would you like to collaborate on? | Environmental Educational Suitcase  *Sa i actiu al meu barri* |
| What can you contribute as a collaborator? What are your knowledge, skills or experience to contribute to the project? | Open question |
| Can you attend all the CSU LAB prototyping workshops?  6th October 18h-20h 14th October 16h-19h 15th October 10h | Yes /No |
| What is your name? | Open question |
| What is your contact e-mail address? | Open question |
