# Supplementary material for: Citizen science and social innovation as citizen empowerment tools to address urban health challenges: The case of the urban health citizen laboratory in Barcelona, Spain
Source: PLoS One. 2024 Mar 13;19(3):e0298749. doi: 10.1371/journal.pone.0298749 (PMC10936789; doi:10.1371/journal.pone.0298749)
Supplement: S7 Table — (DOCX) [file pone.0298749.s007.docx]

**Table S7. List of proposals received in the open call for ideas.**

|  | **Neighbourhood of the proposer** | **Idea** | **Local topic addressed** |
| --- | --- | --- | --- |
| **Online form** | | | |
| 1 | Eixample | *Diagnòstic i actuació urbana* (Diagnosis and urban action) is based on the creation of a commonplace, physical space that is representative of the ideas and proposals of the community (..). The aim of this proposal is to connect the existing community network linked to a tangible, attractive and referential space that serves as a place for dialogue and recreation for new actions in the neighbourhood. | Community networks |
| 2 | Eixample | To understand the social sports infrastructure as a promoter of social relations in the neighbourhood. To this end, it is proposed to improve and think about the spaces currently found in the neighbourhood, as spaces that are a point of reference in the neighbourhood, in which sporting and non-sporting uses are allowed, based on spontaneous sporting activities, understood as mechanisms for participation in the neighbourhood. | Community networks |
| 3 | Guinardó/ Vallcarca | Organise one or more workshops in which, through dialogue, creation and exploration of the body, the connection between human health and planetary health is addressed, and with the so-called ecosomatic methodologies, which explicitly connect body and environment. In addition to the urban gardening activities in Trinitat Vella, these workshops will be aimed at different age groups in order to deepen the dialogue and adjust as much as possible to the needs of each person, also following a protocol of inclusivity for people with functional or intellectual diversity. From the materials resulting from the meetings, an archive of eco-friendly practices will be compiled, an offer from the citizens to the citizens and political leaders of the neighbourhood, on how to intensify individual-collective relations in the urban environments of Trinitat Vella. | Inclusion and social integration  Local biodiversity |
| 4 | Guinardó | *Sa i actiu al meu barri* (Healthy and active in my neighbouhood*)*. Design and implement circuits, challenges, good practices and advice at different points in the neighbourhood through playful and differentiated signage on public roads, which encourages physical activity and a better state of mind, with the aim of reducing obesity and sedentary lifestyles among other associated pathologies, thereby contributing to the physical and mental health of people. | Physical and mental health |
| 5 | Sant Antoni | *DansArt Salut* (DanceArt Health). Integrated dance workshops for all. Movement, expression, creativity and community. With a final show for the neighbours! | Community networks |
| 6 | La Rivera | Smart farm is an authentic farm that can be controlled via the internet. | Community networks |
| 7 | San Gervasi | We propose an activity that aims to provide a space for the community to connect with each other, with the environment and music through a live musical installation that invites you to dance without rules. It is a healthy, family-oriented event that celebrates the encounter, sport and culture. | Inclusion and social integration |
| 8 | Sagrada Familia | Creation of a youth social innovation laboratory through art. A collective of young people where, through artistic and cultural language, the challenges we face in our neighbourhood, region, country and planet (problems included in the SDG agenda, social inclusion, environmental problems, and educational challenges) are put at the centre of our work and together we devise, design and create possible solutions (..). The result of the meetings will be the creation of a work, performance or a concrete act where we will also define an impact measurement tool based on the selected problem. | Inclusion and social integration |
| 9 | Porta | Autochthonous shrubby vegetation in the tree canopies. | Local biodiversity |
|  |  | Urban pedestrian and public and collective transport, cycling and walking plan. | Air pollution |
|  |  | To create more green breathing and recreational areas that inspire the development, with the support of official and of private entities and neighbourhood residents, of different leisure, environmental and communication activities, as well as others inspired by the arts: storytelling, theatre, dance, painting, etc. | Inclusion and social integration |
| 10 | Trinitat Vella | This neighbourhood is being used as a car park for the people of the northern part of Barcelona, the green zone ends before entering the neighbourhood, and the residents have to pay for a car park to be able to park. For all these reasons we demand equal treatment with other citizens, and that the green zone be extended to the whole neighbourhood. | Urban mobility |
| 11 | Trinitat Vella | Our proposal aims to educate and raise awareness in the knowledge and care of the environment among children and young people in the schools of Trinitat Vella by means of an educational suitcase or educational kit for monitoring and analysing environmental pollution indicators in Trinitat Vella.  Suitcase with a sample collection device to work on the monitoring of pollution indicators and an educational guide for teachers. | Air pollution |
| 12 | Trinitat Vella | My proposal is to be able to carry out activities with the elderly, as the elderly have been left on the side, but we must take them into consideration as they have been the people who have taught us to grow and it would be a good idea to be able to do things with the elderly. | Community networks |
| **Park(ing)Day** | |  |  |
| 13 | Trinitat Vella | A road can be closed 1 day a week and activities can take place | Urban mobility |
| 14 | Trinitat Vella | Bike lane designed for children and adults (family) | Urban mobility |
| 15 | Trinitat Vella | A car-free pedestrian day | Urban mobility |
| 16 | Trinitat Vella | Adapted bike lane + bike stops | Urban mobility |
| 17 | Trinitat Vella | Solar panels with public money in all squares to illuminate all public buildings | Air pollution |
| 18 | Trinitat Vella | Better insulate roads | Air pollution |
| 19 | Trinitat Vella | Remove the *chiringuitos* from the Trinitat Vella bridge (to the new blocks) for more air | Air pollution |
| 20 | Trinitat Vella | Free neighbourhood activities for different age groups | Inclusion and social integration |
| 21 | Trinitat Vella | Insect hotels in the park | Local biodiversity |
| 22 | Trinitat Vella | Vertical gardens | Local biodiversity |
| 23 | Trinitat Vella | Children's playgrounds and electric cars | Local biodiversity |
| 24 | Trinitat Vella | More safety for children | Community networks |
| 25 | Trinitat Vella | Skate Park | Community networks |
| 26 | Trinitat Vella | Implement green areas for puppies | Community networks |
