# Supplementary material for: Citizen science and social innovation as citizen empowerment tools to address urban health challenges: The case of the urban health citizen laboratory in Barcelona, Spain
Source: PLoS One. 2024 Mar 13;19(3):e0298749. doi: 10.1371/journal.pone.0298749 (PMC10936789; doi:10.1371/journal.pone.0298749)
Supplement: S8 Table — (DOCX) [file pone.0298749.s008.docx]

**Table S8. Co-created communication plan.**

|  |  | **Public** | | | | |
| --- | --- | --- | --- | --- | --- | --- |
| **Item** |  | Children | Teenagers | Neighbours and citizens | Administration | Scientific community |
| Air pollution and noise data |  |  |  | Interview on Trini Jove's radio station | Meeting with local stakeholders | Scientific article |
|  |  |  |  | Contact with local television Betevé |  |  |
|  |  |  |  | Press release |  |  |
| Methodological framework |  | Draft project and seek calls for proposals to give continuity to the project in the territory | | |  |  |
| Prototypes: urban signalling kit & educational environmental suitcase |  | Training for the neighbourhood's educational space: Esplai La Tortuga and Aula Ambiental de Sant Andreu | | Informative walk through the neighbourhood |  |  |
|  |  | To make educational resources known to the educational resource centre of Catalonia (CESIRE) | |  |  |  |
| Video - documentary of the process |  | Social media outreach \| Submission to contests | | | | |
|  |  |  | | | | |

Items, target audience and agreed actions to disseminate the project and its results.
