# Supplementary material for: Citizen science and social innovation as citizen empowerment tools to address urban health challenges: The case of the urban health citizen laboratory in Barcelona, Spain
Source: PLoS One. 2024 Mar 13;19(3):e0298749. doi: 10.1371/journal.pone.0298749 (PMC10936789; doi:10.1371/journal.pone.0298749)
Supplement: S9 Table — (DOCX) [file pone.0298749.s009.docx]

**Table S9. Evaluation online survey.**

| **Question** | **Answer options** |
| --- | --- |
| Q1. What has been your involvement in the project? | 1- I have been aware of the project  5- I have been involved in the whole process |
| Q2. Do you think you have gained any new knowledge about the impact of urbanism on human health? | No / Some new knowledge / I have learnt quite a lot of things |
| Q3. What topics would you say you have learned about that you didn't know about?  *several possible answers | Noise  Air pollution by particles or gases  Temperature  Green zones  Blue zones  Urban mobility  Biodiversity |
| Q4. Do you think you have changed your perception of how urban planning influences your health? | Not at all, I was already fully aware  Yes, I imagined something but the project has helped me to go deeper  A lot! It has raised an issue that I hadn't thought about before |
| Q5. Do you think you have gained new knowledge about how research is conducted? Research question posing, organisation of the work, taking measurements, data analysis, etc. | Yes  No  Other |
| Q6. Have you encountered any new digital tools in the process? | None / 1 / 2 / 3 / 4 / 4+ |
| Q7. Based on the results of the project (data, prototypes, communication, etc.), do you think they will be useful to improve life in the neighbourhood? | Open answer |
