# Supplementary figures and images for: Citizen science and social innovation as citizen empowerment tools to address urban health challenges: The case of the urban health citizen laboratory in Barcelona, Spain
Source: PLoS One. 2024 Mar 13;19(3):e0298749. doi: 10.1371/journal.pone.0298749 (PMC10936789; doi:10.1371/journal.pone.0298749)

**S1 Fig. Participatory diagnosis canvas.**


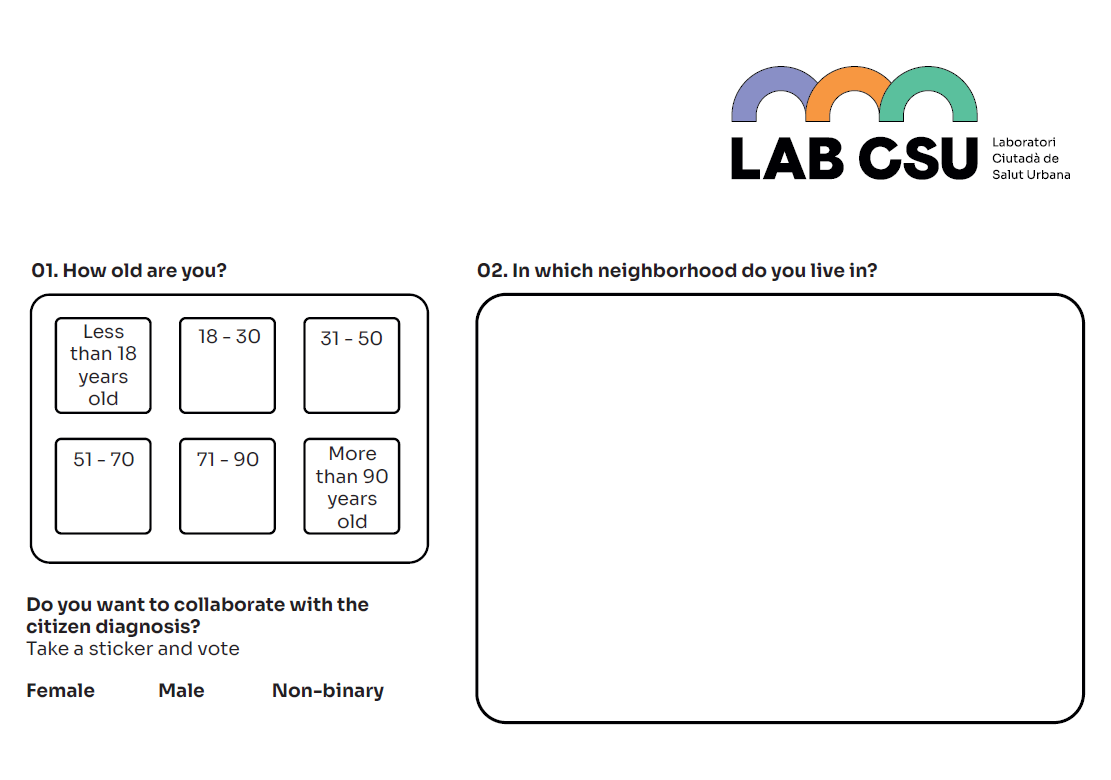


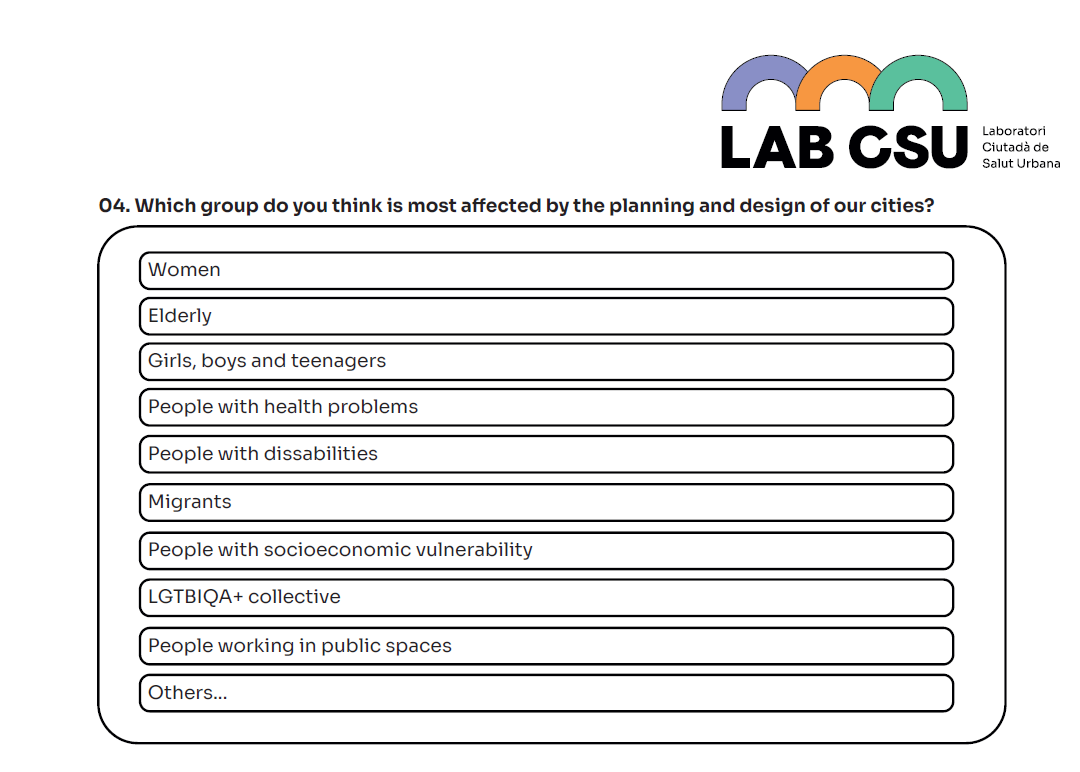


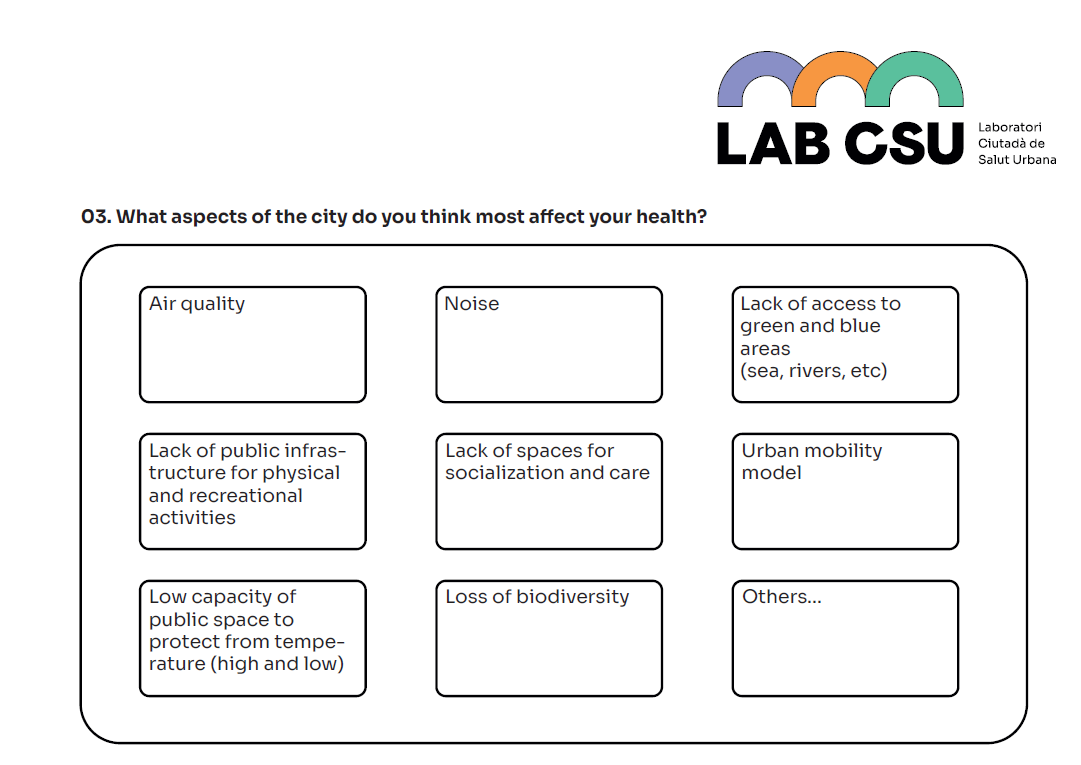

Supplement: S1 Fig — (DOCX) [file pone.0298749.s010.docx]

**S2 Fig Canvas to reflect on the impact of urban planning on human health.**

**
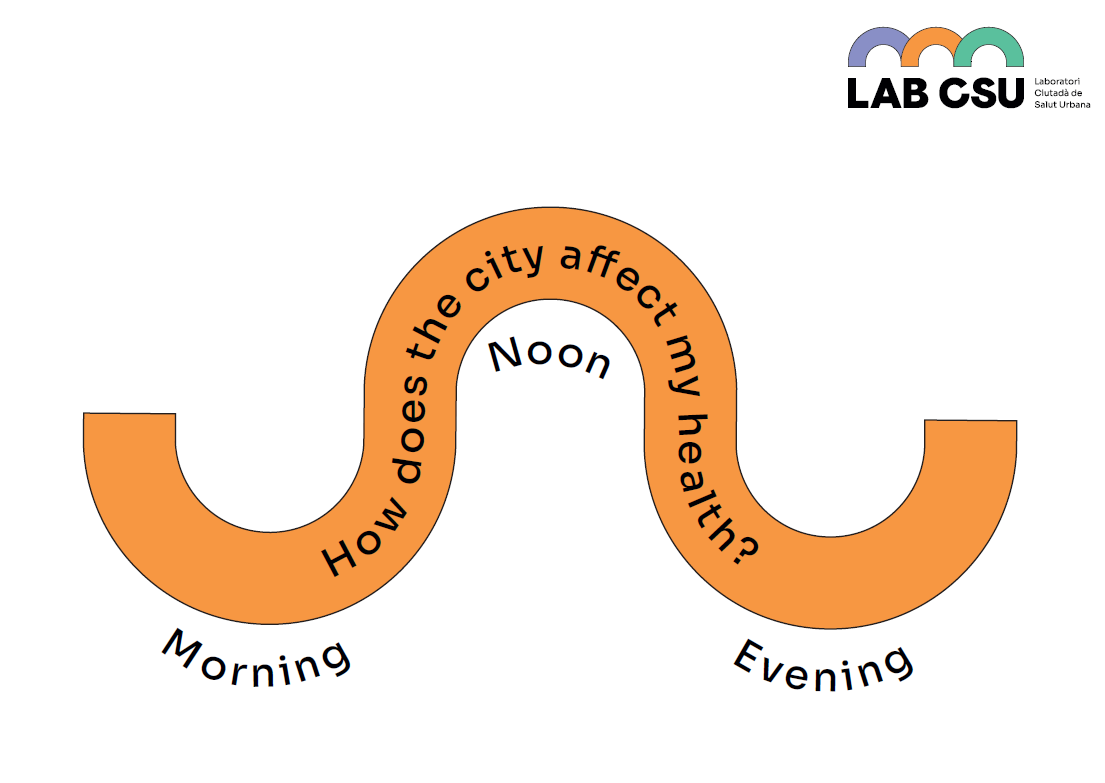
**

Supplement: S2 Fig — (DOCX) [file pone.0298749.s011.docx]

**S3 Fig. Canvas to reflect on the individual's impact on the city.**


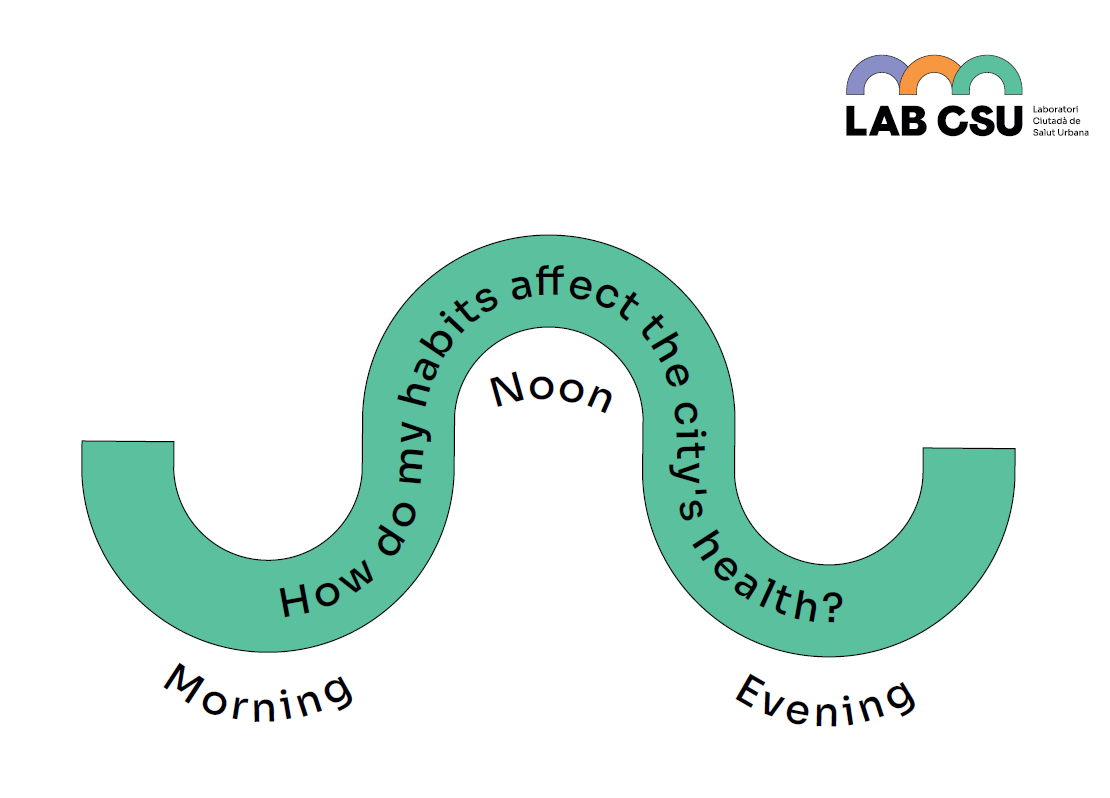

Supplement: S3 Fig — (DOCX) [file pone.0298749.s012.docx]

**S4 Fig. Problem definition canvas.**


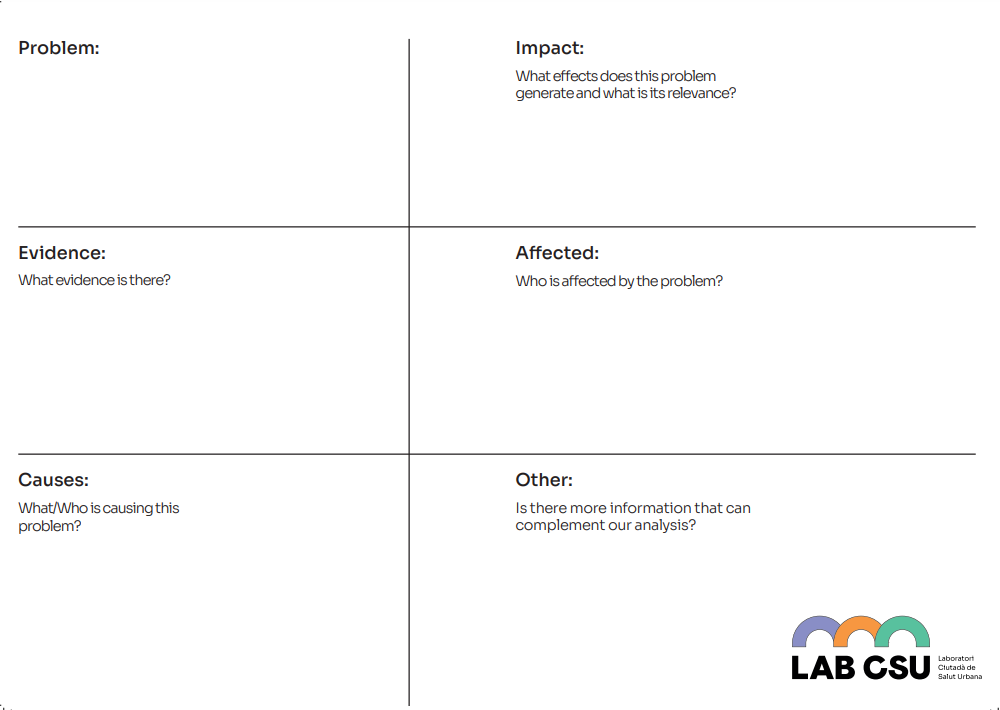

Supplement: S4 Fig — (DOCX) [file pone.0298749.s013.docx]

**S5 Fig. Hypothesis and research question definition canvas.**

**
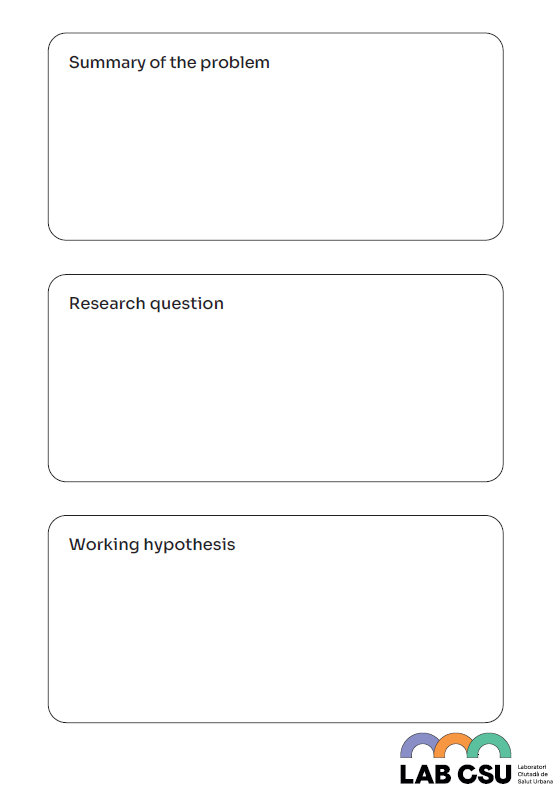
**

Supplement: S5 Fig — (DOCX) [file pone.0298749.s014.docx]

**S6 Fig. Work organization canvas.**

**
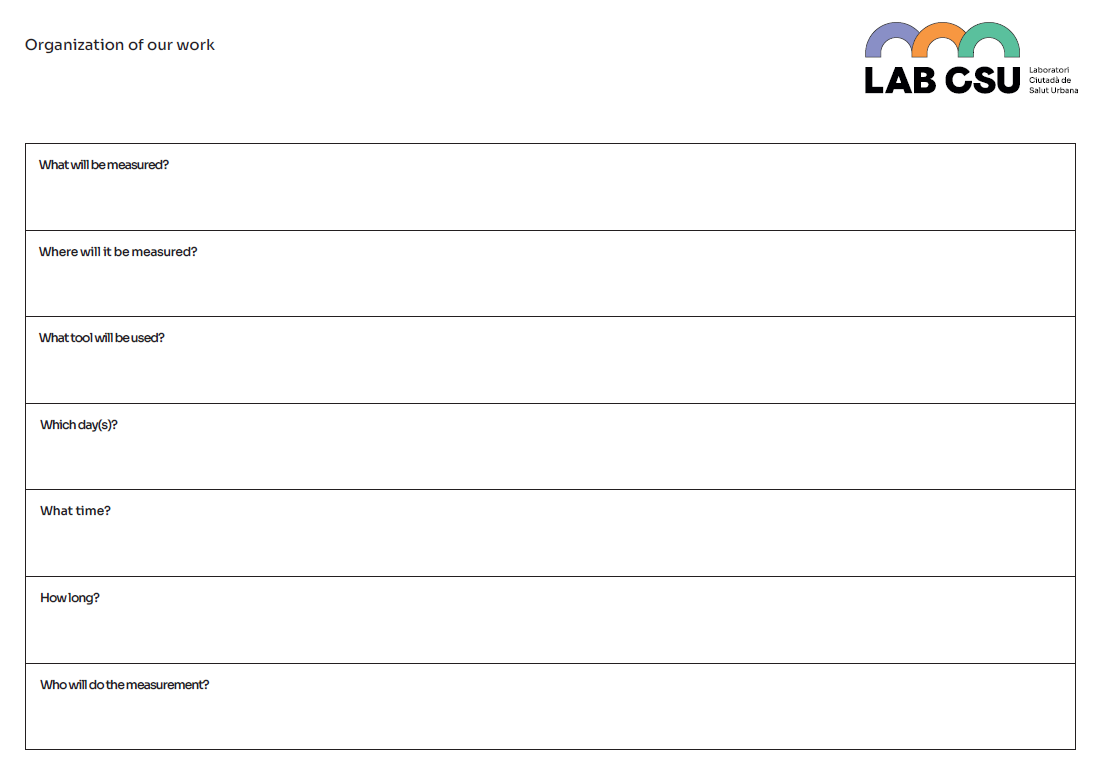
**

Supplement: S6 Fig — (DOCX) [file pone.0298749.s015.docx]

**S7 Fig. Knowledge map canvas.**


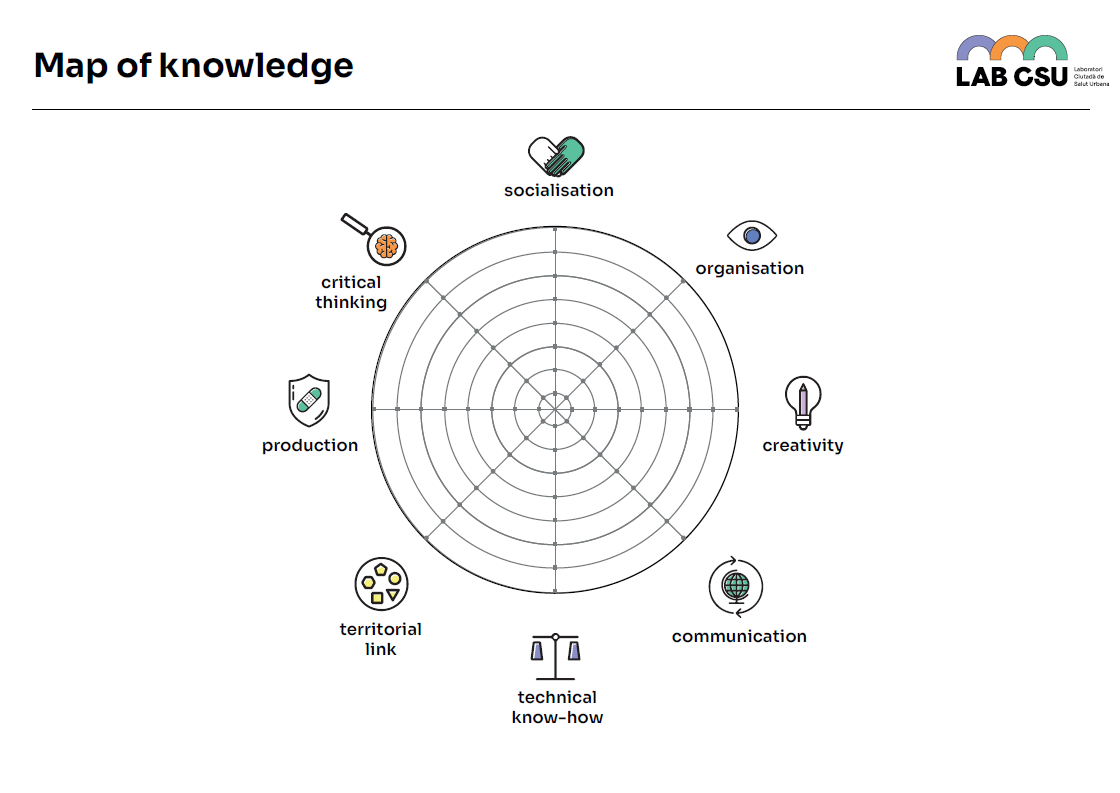

Supplement: S7 Fig — (DOCX) [file pone.0298749.s016.docx]

**S8 Fig.Prototype ideation canvas.**

**
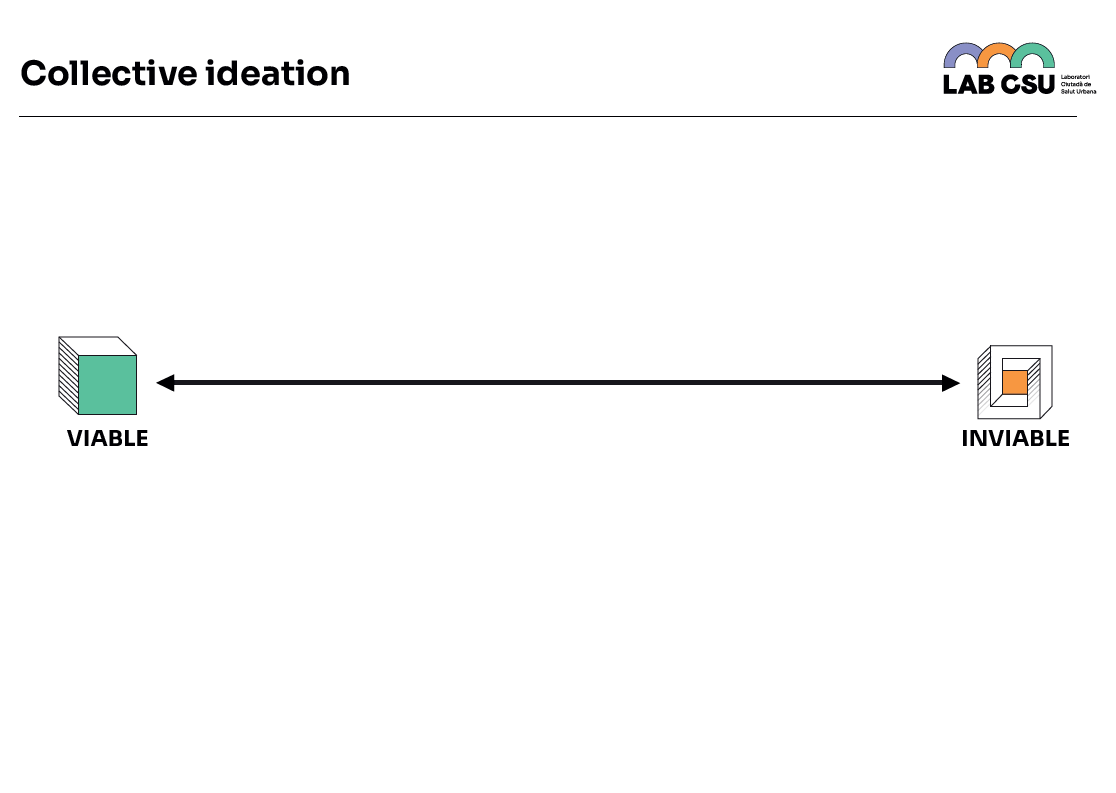
**

Supplement: S8 Fig — (DOCX) [file pone.0298749.s017.docx]

**S9 Fig. Prototype planning canvas.**

**
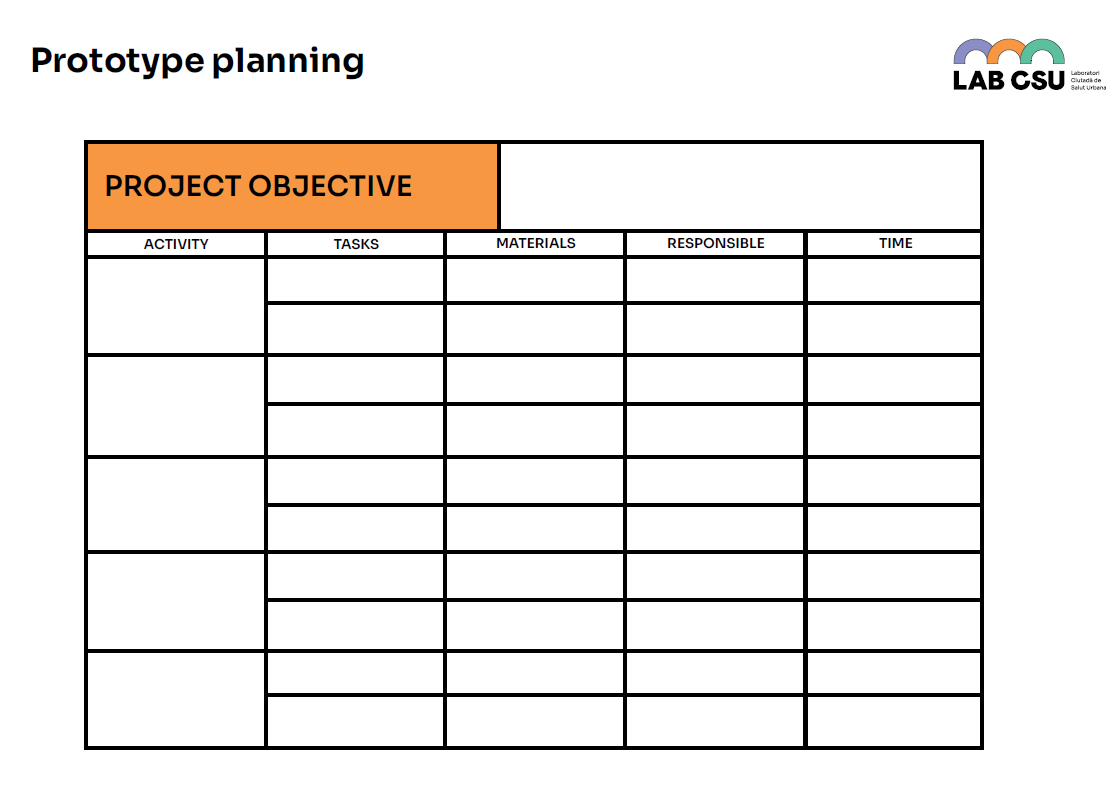
**

Supplement: S9 Fig — (DOCX) [file pone.0298749.s018.docx]

**S10 Fig.. Communication plan design canvas.**


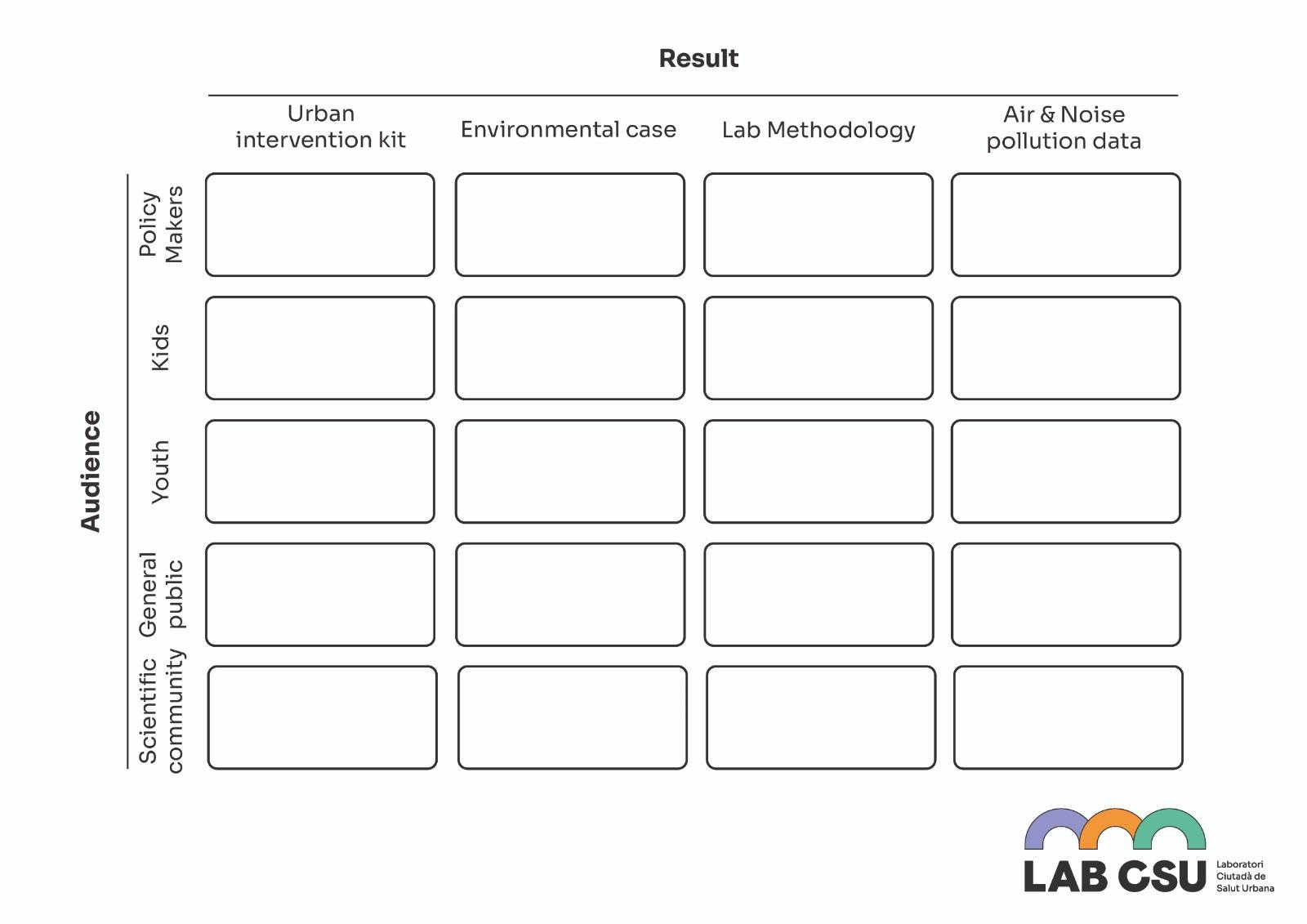

Supplement: S10 Fig — (DOCX) [file pone.0298749.s019.docx]
